# Increasing Biochar Yield in Pyrolysis by Adding Ash

**DOI:** 10.1021/acs.est.5c14739

**Published:** 2026-08-04

**Authors:** Jannis Grafmüller, Michael Tobler, Philipp Vögelin, Thomas D. Bucheli, Hans-Peter Schmidt, Nikolas Hagemann

**Affiliations:** † Ithaka Institute, 63773 Goldbach, Germany; ‡ Institute of Sustainable Energy Systems (INES), 589822 Offenburg University, 77652 Offenburg, Germany; § Holz & Forst Consulting GmbH, 4102 Binningen, Switzerland; ∥ 468899 Industrielle Werke Basel (IWB); Margarethenstrasse 40, 4002 Basel, Switzerland; ⊥ Environmental Analytics, 87635 Agroscope, 8046 Zurich, Switzerland; # Ithaka Institute, 1974 Arbaz, Switzerland

**Keywords:** alkali and alkaline earth metals, AAEM, catalysis, nutrient recycling, PyCCS

## Abstract

Wood ash additives increase biochar yield in pyrolysis via catalysis by alkali and alkaline earth metals. However, it remains unclear how ash and biomass properties govern the magnitude of this catalytic effect. Here, we pyrolyzed pelleted and ash-amended woody and gramineous biomasses at 500–550 °C. At pilot scale, 5% (w/w) amendment of different ashes increased dry and ash-free biochar yield (*y*_daf_) by 13–37% and carbon yield (*y*_C_) by 2–33% for woody feedstocks and decreased yields by 5–6% for grass or straw. Accordingly, mixing grass with softwood at increasing ratios reduced the effects of added ash. The yield increase depended on biomass type rather than on the intrinsic ash content of the biomass, as positive effects were observed even for woody feedstocks with a relatively high ash content, unlike gramineous biomass. Yield increases observed for different added ashes did not correlate with ash properties, pending further investigation. Toxic hexavalent chromium from ash additives was not detected in biochar, suggesting its reduction to nontoxic Cr­(III) during biochar production. At industrial scale, *y*_daf_ increased by 11% and *y*_C_ by 6% through adding 4% ash to landscaping wood, demonstrating the scalability of wood ash use. Adding ash to woody feedstock increased biochar production, pyrolysis economy, and carbon sink potential.

## Graphical abstract

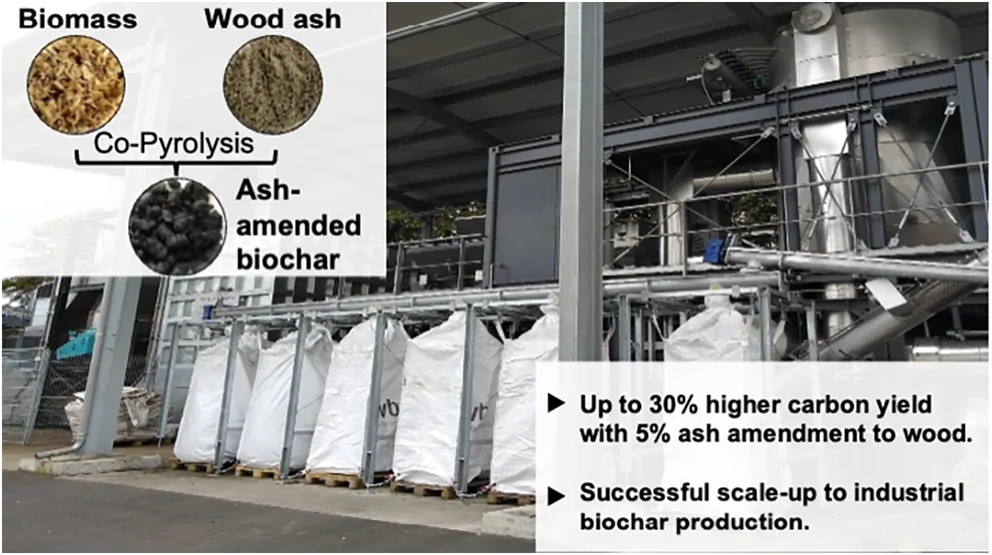

## 1 Introduction

Biomass pyrolysis and nonoxidative use of biochar are the key elements of the carbon dioxide removal strategy called pyrogenic carbon capture and storage (PyCCS).^1^ The removal of CO_2_ from the atmosphere occurs during photosynthesis, so that the climate impact of this approach is determined in the long term by both the availability of biomass and the yield of biochar from pyrolysis. Biomass is a limited resource due to the availability of land for cultivation, suitable climate, and competition with food production and other sustainability goals.^2,3^ Therefore, optimizing biochar yields is essential to efficiently use the available biomass.

In the pyrolysis of lignocellulosic biomass, alkali and alkaline earth metals (AAEM), most importantly calcium (Ca), magnesium (Mg), potassium (K), and sodium (Na), are known to catalyze the pyrolysis process, leading to higher yields of char and pyrolysis gas while reducing the yield of liquid products, by affecting both primary and secondary pyrolysis reaction pathways.^4–6^ Alkali and alkaline earth metals can be deliberately added to biomass prior to pyrolysis via different types of materials, including wood ash.^4,7–10^ Adding wood ash in biomass pyrolysis also adds the macronutrient K, as well as other nutrients, in a plant-available form to biochars, helping to close AAEM nutrient cycles.^8,9,11^ Using biomass ash in biochar production could synergistically link PyCCS with Bioenergy Carbon Capture and Storage (BECCS), which includes biomass combustion, resulting in nutrient-rich ashes to be disposed of.^11^ However, knowledge is limited on how different ashes alter biochar yields and properties when blended with biomass feedstocks.

To evaluate the effect of ash additives on biomass pyrolysis, the mass yield (*y_M_*) of biochar production is not expedient (see eq 2 in the Supporting Information-SI). Therefore, the dry and ash-free biochar yield (*y*_daf_) and biochar carbon yield (*y*_*C*_) of pyrolysis have been investigated.^8,9^ For *y*_daf_, the biochar mass, minus its ash and water content, is related to the feedstock mass minus its ash and water content (see eq 3 in the SI). For the calculation of *y*_*C*_, the mass of total carbon in the biochar is related to the total carbon present in the feedstock before pyrolysis (eq 4 in the SI).

For woody biomass, adding ash prior to the pyrolysis increased *y*_daf_ by up to 40–90%.^8,9^ This was achieved with ash amendments of up to 40–50%; however, ash was most effective when added at a concentration of 5–10% (w/w) to softwood.^9^ Yield increases per amount of added ash were lower for amendments higher than 20%.^9^ Further, adding ash to ash-rich biomass had no effect on *y*_daf_ for biogas residue and even decreased it for maize silage at a pyrolysis temperature of 500 °C.^12^ These previous studies have each tested only one combination of biomass and ash. There has been no systematic comparison of blending different biomasses or ashes. Yet, combining this limited existing data allows to conclude that the effect of an ash amendment on *y*_daf_ and *y*_*C*_ might vary with biomass and ash type, and can even have no or negative effects when a biomass with, e.g., high absolute intrinsic ash content, such as straw, is pyrolyzed. The ash composition could further be of importance, since different AAEM may differently impact the catalyzing char formation in biomass pyrolysis.^5^ Ash-induced changes of biochar yield were also shown to be temperature-dependent. Dry and ash-free biochar yield was less pronounced at 400 °C than at 425–500 °C.^9^ For pure potassium doping, the increase in carbon yield peaked at 450–550 °C and declined at higher temperatures, suggesting that 500 °C represents a near-optimal pyrolysis temperature for maximizing the carbon yield benefit of AAEM addition.^7^ Besides AAEM, biomass ash contains potentially toxic elements (PTE),^13^ which may limit compliance with fertilizer regulations and thus its direct soil application. One of these PTE is chromium, in particular its hexavalent speciation (Cr­(VI)),^14^ which is highly toxic and carcinogenic.^15^ Bottom ashes can be enriched in Cr­(VI),^13^ due to the low volatility of Cr and the oxidative atmosphere in combustion systems. The reductive atmosphere in pyrolysis reactors is supposed to reduce Cr­(VI) to less-toxic Cr­(III), which has not been explored systematically yet.

On pilot-plant scale, we first varied the biomass composition by mixing low-ash softwood with ash-rich grass to study the effect of an ash amendment on *y*_daf_ and *y*_*C*_ in dependence on biomass properties. In addition, four further biomasses were used to test a broader range of properties. Second, we tested four different wood ashes and one gasifier coke with a high ash content to link their individual AAEM contents, solubility, and speciation with their effects on softwood-biochar production. Detailed analyses of the ashes can be found in Section 2.1 in the SI. Third, we tested whether Cr­(VI) originating from an ash additive remains inert or is reduced during pyrolysis. Fourth, we applied wood ash in biomass pyrolysis at an industrial scale (300 kg biomass input per hour) to quantify effects on *y*_daf_ and *y*_*C*_. With this study, we aim to gain more knowledge on the mechanisms and overall potential of ash-induced increases in *y*_daf_ and *y*_*C*_ of pyrolysis products and intend to provide practical recommendations for the biochar industry.

## 2 Materials and Methods

### 2.1 Sampling of Ash Additives

Five different ashes from wood-fired power plants in Switzerland were sampled by the plant operators and labeled according to their origin: Sissach, Brislach, Möhlin, Gruyère, and Zeglingen. The Zeglingen sample was an ash-rich gasifier coke from a combined heat and power unit, while all other samples were bottom ashes from burning chambers. All systems processed untreated Swiss forest wood. The ash from Gruyère was used for pyrolysis at both the pilot-plant and industrial scales, obtained from two different sampling dates, which were approximately two months apart. The Gruyère sample used for pyrolysis at industrial scale is referred to as “Gruyère_large”. The ashes were sieved to a particle size of <2 mm to separate stones, residual char pieces in the gasifier coke, and other artifacts (Table S1 in SI).

### 2.2 Biomass Selection and Pelletization for Pyrolysis on Pilot-Plant Scale

The following six biomasses were used as feedstock for biochar production (elemental composition displayed in Table S6): commercial softwood bedding (GermanHorse, AllSpan, Karlsruhe, Germany), mixed Swiss forest wood, grass (long grass from an urban area managed with single-cut mowing), wheat straw, conifer bark mulch (both from Landi Schweiz AG, Dotzingen, Switzerland), and waste timber category A2/A3 (Lindum AS, Norway, cf. Sørmo et al.^16^).

Note that all pilot-scale feedstocks – both controls and ash-amended samples – were pelleted. The pelleting benefit, i.e., the impact of pelleting on biochar yield, quantified separately in Figure S7, is thus equally present in both treatments, and the yield differences reported here reflect the effect of ash addition independent of the pelleting effect. Forest wood, grass, wheat straw, and waste timber were first milled with a hammer mill to <12 mm (HM420B, EverTec, Groß-Zimmern, Germany). Softwood bedding and bark were obtained ready for pelletization. Pellets with a diameter of 6 mm were produced using a pellet press (WK230, EverTec, Groß-Zimmern, Germany) after homogenization of the respective biomass with the ash additive and water to achieve a water content of 25% (w/w). Obtained pellets were dried to 5–15% (w/w) residual water content at 40 °C for 12 h, sieved to >6 mm, and stored at room temperature prior to pyrolysis (∼7 days storage time). In total, 22 sets of pellets from different feedstock blends were prepared (Table S2).

### 2.3 Pyrolysis at Pilot-Plant Scale

Pyrolysis experiments on pilot-plant scale were sequentially performed on a PYREKA research pyrolysis unit (Pyreg GmbH, Dörth, Germany), a continuously operated, electrically heated auger reactor.^17^ The reactor was purged with 2 L min^–1^ N_2_ that passed through the reactor (8 cm diameter) concurrently with solids. The biomass was fed continuously via a rotary valve, and the biochar was collected in a water-cooled container that was emptied intermittently. Pyrolysis was performed at the highest treatment temperature (HTT) of 500 °C with a feedstock residence time (RT) of 10 min in the reactor. After a stabilization time of five RT, during which the product was discarded, biochar was sampled without replicates for a total of three RT.

### 2.4 Pyrolysis at Industrial Scale

Industrial-scale experiments were carried out on a PX1500 (Pyreg GmbH, Dörth, Germany) operated by Industrielle Werke Basel (IWB, Basel-Kleinhüningen, Switzerland).^18^ The reactor had a biomass throughput of 300 kg h^–1^ or 2200 t year^–1^ (dry matter). This pyrolysis plant consists of two horizontally mounted double screw reactors that are supplied with biomass from a storage tank via rotary feeders. The reactors are double-walled and heated indirectly by hot exhaust gases from pyrolysis gas combustion (Figure S1). The pyrolysis was conducted with a RT of 20 min and an HTT of 550 °C. The pyrolysis reactors were continuously fed with predried landscaping wood (elemental composition: Table S6). The storage container was filled with biomass from the bunker at 15 min intervals via a conveyor belt, which took a minute each time. The biomass input over time to the reactors was determined by recording the load cell of the storage tank over time and was obtained as an averaged value over the whole experimental runtime (Figure S2). The wood ash (sample Gruyère_large) was scattered into the biomass stream each time the storage tank was automatically filled (5 kg of ash per fill with 90–100 kg dry biomass, Figure S3), which resulted in 4% (w/w) ash amendment. After an initial process stabilization time of four RTs (4 min × 20 min), biochar samples were taken. For each sample, biochar was sequentially filled for 6 min in a separate 15 kg bag, which was weighed to 0.1 kg accuracy to determine biochar mass flow. This process was performed nine times for both the control biochar (no ash amendment) and the ash-amended biochar. From these nine subsamples, three subsamples were combined three times to form a total of three subsamples. These three combined samples were then representatively sampled,^19^ so that three representative biochar samples per treatment were analyzed.

### 2.5 Biomass, Ash, and Biochar Analysis

Ash, biomass, and biochar analyses were conducted by Eurofins Umwelt Ost GmbH (Bobritzsch-Hilbersdorf, Germany). Basic ash, biomass, and biochar analyses were conducted following international guidelines, as detailed in Table S3 in the SI. Soluble AAEM contents in the ashes were quantified following DIN EN ISO 17294–2 in aqueous eluates prepared by shaking horizontally at 150 rpm for 1 h at a solid:liquid ratio of 1:10.^20^ Quantitative X-ray diffraction analysis (XRD) of the ashes was performed by QMineral (Heverlee, Belgium).

### 2.6 Data Analysis

Biochar yields (*y*_*M*_, *y*_daf_, and *y*_*C*_) and increase in nitrogen yield (Δ*y*_*N*_) of pyrolysis were calculated according to the literature^9^ as detailed in eqs 2–4 in the SI. Pilot-scale experiments were performed without replicates due to the substantial feedstock quantities required per run and the large number of feedstock combinations tested (*n* = 22). Measurement uncertainty of biochar yield was estimated by error propagation from feedstock mass flow variability (see SI eqs 6–14). This approach provides conservative uncertainty estimates. Results from pilot-scale experiments should therefore be interpreted as indicative trends.

The industrial-scale experiment, which included replicate sampling and full statistical testing, provides a quantitatively robust basis for the scalability of the key findings. Here, nine individual values for *y*_*M*_, *y*_daf_ and *y*_*C*_ per treatment were calculated and statistically evaluated with GraphPad Prism v9.4.1 (one-way ANOVA and the Šídák multiple comparison test, α < 0.05). Significance tests were not applied to yield data from experiments on pilot-plant scale, since biochar sampling was performed with single replicates and, therefore, the biomass mass flow was the only variable contributing to deviations, which was deemed insufficient to perform further statistical analysis. The H/C_org_ molar ratios from biochars produced at pilot-plant scale, with or without an ash amendment, were compared by an unpaired *t* test (α < 0.05). The observed increases of *y*_daf_ and *y*_*C*_ due to ash amendment to varying biomasses were modeled in JMP 18 (SAS Institute, Cary, North Carolina, USA), applying a multivariate regression analysis using the intrinsic lignin, cellulose, hemicellulose, and ash contents of the biomasses as variables. Variables identified as nonsignificant with JMP (*p* > 0.05) were excluded from the model. Linear regression analysis of *y*_daf_, *y*_*C*_, or Δ*y*_*N*_ with ash properties was performed by calculating Pearson correlation coefficients (*r*) with GraphPad Prism.

## 3 Results and Discussion

### 3.1 Impact of Ash Addition on Biochar Production at Pilot-Plant Scale

#### 3.1.1 Effect of Ash Amendment on Biochar Yield of Different Biomasses

Amending softwood with 5% Sissach ash increased *y*_daf_ and *y*_*C*_ by 13% and 9%, respectively (Figure 1a and Table 1). This was also achieved when the ash was added to a mixture containing 25% grass and 75% softwood (Figure 1a). The more grass was added to the softwood, the lower the increase in yields due to the ash amendment. At a grass content of 75%, no substantial yield changes occurred anymore (Figure 1a). For pure grass, the ash amendment reduced *y*_daf_ and *y*_*C*_ by 5% and 6%, respectively, compared to the nonamended grass feedstock (Figure 1a). A similar yield-reducing effect was observed when ash was added to straw feedstock (Figure 1b). Effects on forest wood and bark were comparable to those of softwood, while the ash blending of waste timber at 5% of Sissach ash decreased *y*_daf_ and *y*_*C*_ by 6% and 8%, respectively (Figure 1b). Waste timber was, thus, the only woody feedstock for which a decline in *y*_daf_ and *y*_*C*_ occurred upon the ash amendment. The observed decrease with waste timber feedstock is inconclusive based on our other experiments and literature using woody feedstock,^8,9^ and requires further verification. The waste timber originated from category A2/A3 according to Norwegian standard NS 4414, which encompasses chemically treated wood, potentially including chromated copper arsenate or other preservative treatments as evidenced by the elevated As, Cr, and Cu concentrations detected in the waste timber biochar (Table S6), whose impact on pyrolysis has not been studied. It may also contain plastics and polymer-based binders that in turn may include flame retardants. The exact nature and content of these contaminants was not determined, which limits mechanistic interpretation. Alkali and alkaline earth metals are known catalysts in pyrolytic plastic and polymer recycling,^21^ potentially promoting complete volatilization of plastics already at 500 °C, while 600 °C has been suggested for complete plastic elimination,^22^ thus reducing char yields from this feedstock. We retain this data as a relevant real-world observation, but a dedicated follow-up study with chemically defined “artificial” waste wood (e.g., untreated wood treated with defined preservatives, flame retardants, and/or polymers) would be necessary to mechanistically explain the observed behavior.

**Figure 1:**
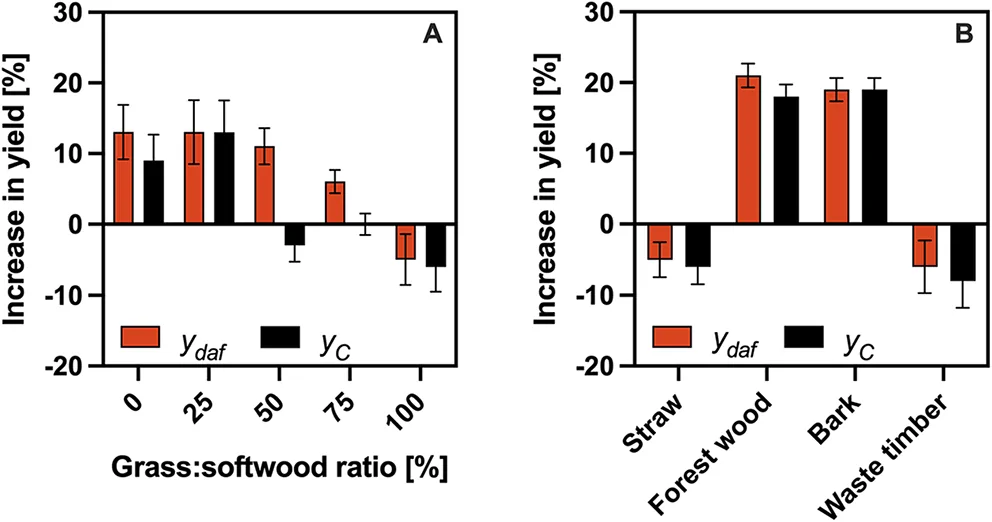
(A) Increases in the yield of dry and ash-free biochar (*y*_daf_) and in carbon yield (*y*_C_) of biochar production due to addition of 5% (w/w) Sissach ash to different mixtures of grass and softwood at the indicated mass ratios. (B) Increases in *y*_daf_ and *y*_C_ due to addition of 5% (w/w) Sissach ash to different biomasses. All biochars were produced at 500 °C with a 10 min residence time in the pilot-scale pyrolysis reactor. Error bars were calculated according to the error propagation law based on the variation of the biomass transport in the pyrolysis unit.

**Table 1:** Biochar Properties: Elemental Analysis (Organic and Total Carbon (C_org_ and C, Respectively), Hydrogen (H), Nitrogen (N), Sulfur (S)), Ash Content, and Molar H/C_org_ Ratio^a^,^b^

biochar	C [%]	*C*_org_ [%]	H [%]	N [%]	ash [%]	H/C_org_	*y*_*M*_ [%]	*y*_daf_ [%]	*y*_*C*_ [%]
Softwood	87	87	2.8	0.2	2.0	0.38	22 ± 1	21 ± 1	38 ± 1
Softwood/Grass (75:25)	79	79	2.8	0.6	8.2	0.42	26 ± 1	24 ± 1	42 ± 2
Softwood/Grass (50:50)	75	75	2.6	0.8	14	0.41	27 ± 1	24 ± 0	42 ± 1
Softwood/Grass (25:75)	72	72	2.0	1.0	19	0.33	29 ± 0	25 ± 0	43 ± 1
Grass	66	66	2.2	1.3	23	0.40	30 ± 1	26 ± 1	43 ± 1
Softwood +5% Sissach	72	72	2.6	0.1	16	0.43	27 ± 1	24 ± 1	41 ± 1
Softwood/Grass (75:25) +5% Sissach	68	68	2.4	0.4	21	0.42	32 ± 0	27 ± 0	48 ± 1
Softwood/Grass (50:50) +5% Sissach	57	56	2.0	0.6	25	0.42	33 ± 1	27 ± 0	41 ± 1
Softwood/Grass (25:75) +5% Sissach	59	59	2.1	0.9	29	0.43	33 ± 0	26 ± 0	43 ± 0
Grass +5% Sissach	56	55	2.0	1.0	33	0.43	32 ± 1	24 ± 1	40 ± 1
Straw	71	69	2.3	0.8	18	0.40	29 ± 0	26 ± 0	46 ± 1
Forest Wood	83	83	2.8	0.3	5.0	0.40	21 ± 0	20 ± 0	35 ± 1
Bark	76	75	2.8	0.9	11	0.44	32 ± 1	31 ± 1	50 ± 1
Waste timber	84	83	3.1	1.7	4.0	0.45	24 ± 1	23 ± 1	41 ± 1
Straw +5% Sissach	62	61	2.0	0.7	27	0.39	30 ± 1	24 ± 1	43 ± 1
Forest Wood +5% Sissach	73	72	2.6	0.2	16	0.43	27 ± 0	24 ± 0	41 ± 1
Bark +5% Sissach	69	68	2.5	0.7	19	0.44	40 ± 0	37 ± 0	59 ± 0
Waste timber +5% Sissach	70	70	2.7	1.2	18	0.46	25 ± 0	19 ± 0	37 ± 1
Softwood +5% Zeglingen	79	78	2.8	0.2	10	0.43	31 ± 0	29 ± 0	50 ± 1
Softwood +5% Möhlin	73	72	2.7	0.3	15	0.45	30 ± 1	27 ± 1	45 ± 1
Softwood +5% Gruyère	71	70	2.9	0.2	16	0.49	31 ± 1	28 ± 1	46 ± 2
Softwood +5% Brislach	70	69	2.7	0.2	15.1	0.47	27 ± 1	24 ± 0	38 ± 1
Softwood +10% Brislach	62	60	2.1	0.3	23.5	0.42	32 ± 1	27 ± 1	42 ± 1
Softwood +20% Brislach	49	45	1.6	0.2	34.6	0.42	47 ± 1	38 ± 1	53 ± 1
Landscaping Wood^b^	72 ± 1	71 ± 1	2.5 ± 0.1	0.8 ± 0.1	14.2 ± 0.1	0.43 ± 0.03	37 ± 1	33 ± 2	53 ± 2
Landscaping Wood +4% Gruyère_large^b^	59 ± 1	58 ± 1	2.2 ± 0.1	1.0 ± 0.1	26.9 ± 0.4	0.46 ± 0.02	48 ± 2	36 ± 2	57 ± 2

a Mass yield of biochar (*y_M_*), dry and ash-free biochar yield (*y*_daf_), and carbon yield of pyrolysis (*y_C_*). The different biomasses were amended with the specified amounts (in %, w/w) of the following ashes, when indicated: Sissach, Zeglingen, Möhlin, Gruyère, Gruyere_large, and Brislach. Biochars were pyrolyzed at 500 °C with a 10 min residence time at pilot-plant scale. Biochars produced from landscaping wood were pyrolyzed on an industrial plant at an estimated 550 °C with 20 min residence time. Errors were calculated according to the error propagation law, unless otherwise specified.

b For biochars produced from landscaping wood, errors indicate the standard deviation of three replicates for elemental analysis and of nine replicates for *y*_*M*_, *y*_daf_. and *y*_*C*_.

The bark had an intrinsic ash content of 6.7% (Table S6), which was similar to grass and straw but considerably higher compared to softwood and forest wood (<1%). Therefore, the comparably high increases in *y*_daf_ and *y*_*C*_ observed for ash-amended bark (Figure 1b) were not expected. This experiment indicated that the ash-induced yield increase is not necessarily limited by the intrinsic ash content of a biomass but might also be linked to other factors related to biomass composition. The observed increases in *y*_daf_ and *y*_*C*_ can be well modeled by applying a multivariate regression using the intrinsic contents of lignin, cellulose, hemicellulose, and ash in the biomasses as input variables (Figure S6). Only the cellulose and hemicellulose contents had a significant impact (*p* < 0.05) in this model; the other variables were therefore withdrawn. However, due to the small number of data pairs (eight pairs; waste timber was excluded due to the above-mentioned reasons), it is not yet possible to reliably determine which biomass components can best be used to approximate the potential effects of added ash on *y*_daf_ and *y*_*C*_. According to this model, biomasses with high cellulose content combined with low hemicellulose content will respond most positively to an ash amendment, at least for Sissach ash. The results must be further validated with more biomass feedstocks, but also with pure lignin, cellulose, and, e.g., xylan (representative for hemicellulose) amended with ash, to gain a more mechanistic understanding.

A key knowledge gap is understanding why increases in *y*_daf_ and *y*_*C*_ became negative when ash-rich straw and grass samples were amended with additional ash, rather than remaining constant. It could be due to higher biochar reactivity in the pyrolysis reactor and gasification reactions catalyzed by AAEM, particularly by K, when interacting with gasification agents like CO_2_ during pyrolysis.^23–25^ Other than that, gramineous biomasses have a high intrinsic ash content dominated by silica and potassium silicates. Their interaction with alkali metals in ash additives might lead to different effects on the pyrolysis process. Future work should include mineralogical analysis (e.g., XRD) of the biomass intrinsic ash fractions, in our case specifically of the gramineous ones and investigation of the interaction between intrinsic and added AAEM species at different pyrolysis temperatures to elucidate this mechanism.

#### 3.1.2 Effect of Different Ashes Amended to Softwood on Biochar Yield

For all five ashes amended to softwood at 5% (w/w), *y*_daf_ and *y*_*C*_ increased in the range of 11–37% and 2–33%, respectively, compared to nonamended softwood (Figure 2). Using the Brislach ash at a rate of 5% led to the lowest yield increases, while the highest increases were obtained with the Zeglingen gasifier coke. With the Gruyère and Möhlin ash, yield increases were in a similar range as with the Zeglingen ash. In particular, the high yield increases obtained with the Zeglingen gasifier coke ash are promising, as gasifier coke can contain relevant amounts of polycyclic aromatic hydrocarbons and is mostly not allowed for direct soil application unless properly treated beforehand, e.g., by co-pyrolysis.^26^

**Figure 2:**
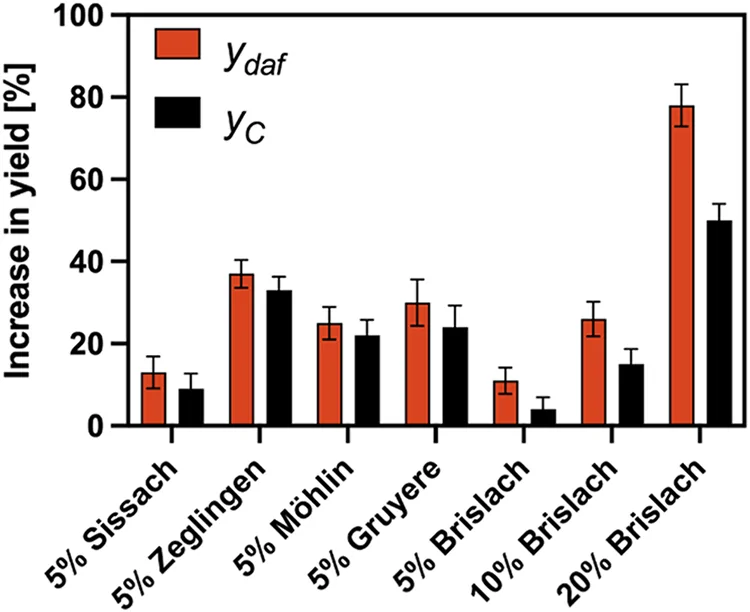
Increases in the yield of dry and ash-free biochar (*y*_daf_) and in carbon yield (*y_C_*) of biochar production due to the addition of different wood ashes to softwood at the indicated ratios (w/w). All biochars were produced at 500 °C with a 10 min residence time in the pilot-scale pyrolysis reactor. Error bars were calculated according to the error propagation law based on the variation of the biomass transport in the pyrolysis unit.

Interestingly, yield increases observed with the different ashes were not significantly correlated to individual AAEM contents or their sum in the ash additives. Wet impregnation of biomass with AAEM additives is generally more effective in enhancing biochar yield than dry mixing.^5^ In our study, moistening the dry mixture of biomass and ash to a water content of 25% before pelleting may have partially solubilized AAEM, improving its catalytic activity. However, *y*_daf_ and *y*_*C*_ were not significantly correlated to the absolute soluble amount of AAEM from the ashes, despite the highest absolute soluble content of AAEM in the Zeglingen and the lowest content for the Sissach samples (Table S5), in line with their contrasting effects on *y*_daf_ and *y*_*C*_.

Melting of AAEM-containing minerals was shown to increase biochar yields by trapping pyrolysis gases inside the solid particles, which leads to higher transformation rates of biomass to biochar.^27^ However, this was probably irrelevant in our study, as wood ashes have melting points well above the pyrolysis temperature of 500 °C used in the present study (1000 °C).^28,29^

Lastly, the speciation of AAEM in the ash might be decisive for its catalytic activity, which was investigated with XRD analyses (Figure S4 and Chapter 2.1 in the SI). The Sissach sample had the highest absolute content and relative share of AAEM in the form of dicalcium silicate (Figure S4), where Ca is allocated within the crystalline structure, leading to an assumed low interaction with the surrounding solid and gas phase during pyrolysis, which could explain the low effect observed for this ash. The Zeglingen sample, in contrast, had no AAEM bound in silicates but the highest absolute and relative contribution of AAEM bound in carbonates, especially CaCO_3_, where Ca is assumed to be better accessible for interaction. Still, correlations of *y*_daf_ and *y*_*C*_ with AAEM speciation, either as carbonates, phosphates, sulfates, oxides, hydroxides, or silicates in the ashes, did not lead to any significant linear correlation.

In summary, no significant linear correlations between ash properties and yield changes were identified. However, with *n* = 5–6 data points, the statistical power to detect moderate correlations is limited. Future studies with a larger number of characterized ashes would be needed to more conclusively evaluate whether specific ash properties predict yield changes. Further, to establish a more mechanistic understanding of this and to identify causalities instead of correlations only, we recommend performing pyrolysis experiments using pure minerals as additives to predict the catalytic activity of complex mineral mixtures, such as wood ash, in biomass pyrolysis. So far, the effect of a specific ash on biochar yields can be estimated at best, if at all, by means of a solubility analysis, as seen for the Zeglingen sample.

The increases in *y*_daf_ and *y*_*C*_ were also dependent on the amount of ash added. Amending the Brislach ash at a rate of 10% to the softwood increased *y*_daf_ and *y*_*C*_ by 26% and 15%, respectively, while at an amendment rate of 20%, increases of 78% and 50%, respectively, were achieved (Figure 2). This dosage-dependent increase in *y*_daf_ and *y*_*C*_ is in line with the literature.^8,9^ The disproportionately higher yield increase at 20% ash amendment compared with 5% and 10% may reflect a threshold effect in AAEM catalysis. At lower ash concentrations, AAEM supply may be the rate-limiting factor for char-forming reactions. At higher ones, higher biochar yields might be related to lower heating rates inside biomass particles due to the alteration of heat transfer by the ash phase. Further mechanistic studies using controlled AAEM dosing and in situ reactor monitoring would be needed to test these hypotheses.

#### 3.1.3 H/C_org_ Ratio and Increase in N Yield of Biochars

The H/C_org_ molar ratio of biochars produced at pilot-plant scale was not significantly changed by an ash amendment, where a decline in *y*_daf_ was observed. When *y*_daf_ increased upon ash amendment, H/C_org_ molar ratios were significantly higher for ash-amended biochars compared to nonamended biochars (Figure 3A). This indicates that a lower share of C_org_ was speciated as aromatic carbon in these biochars produced with ash amendments and/or that the degree of condensation and, with that, the average size of aromatic clusters was reduced.^30^ Still, this needs to be interpreted with caution since H/C_org_ molar ratios are only a proxy and not a direct measure for biochar aromaticity.^30^ The H/C_org_ molar ratio serves as a widely applied proxy for biochar permanence. However, the hydrogen content in biochar is determined by elemental analysis, which quantifies both organic-bound as well as mineral-bound hydrogen and cannot distinguish between them. Thus, an increase in the H/C molar ratio could be explained by the presence of mineral-bound hydrogen, like hydroxides, e.g., of magnesium, calcium, or potassium, or hydrates, e.g., of potassium carbonate. This could be verified, e.g., by elemental analysis after removing the ash content with hydrofluoric acid. However, we are unaware of any established methods to do so. Method development to this end is of utter importance but was beyond the scope of the present study. At the same time, hydropyrolysis or the benzenepoly­(carboxylic acid) (BPCA) method could be used as ash- and H/C-independent proxies for biochar carbon speciation.^31,32^ The increase in H/C_org_ molar ratios of ash-amended biochars is not in line with a previous study.^9^ Still, irrespective of the ash amendment, all biochars had H/C_org_ molar ratios well below the EBC limit of 0.7.^33^

**Figure 3:**
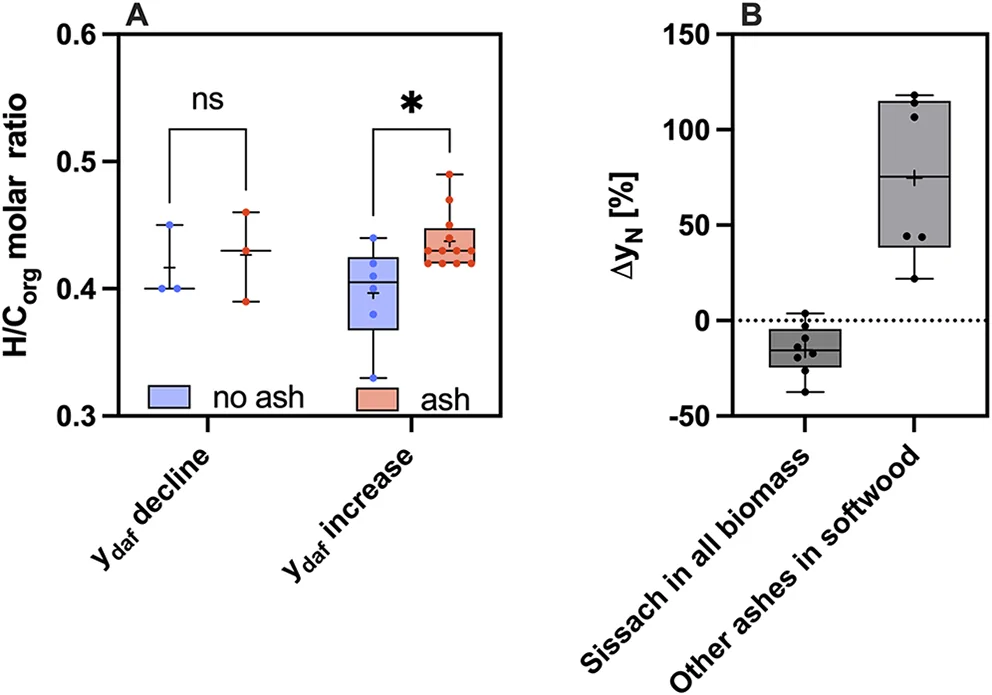
(A) Hydrogen to organic carbon molar ratio (H/C_org_) of all biochars produced on pilot-plant scale without or with an ash amendment to feedstocks separated into two groups: biomasses for which a decline or an increase in dry and ash-free biochar yield (*y*_daf_) due to ash amendment was observed. (B) Increase in nitrogen yield (Δ*y_N_*) due to amendment of 5% (w/w) Sissach ash to all tested biomasses and due to amendment of other ash samples at 5–20% (w/w) to softwood. All biochars were produced at 500 °C with 10 min residence time in the pilot-scale pyrolysis reactor. Error bars indicate the range of minimum to maximum observation; the “+” in boxplots indicates the mean values, and asterisks above error bars indicate a significant difference between treatments in panel (A) (*p* < 0.05, unpaired *t* test, ns: not significant).

The nitrogen yield of biochar production tended to decrease under the amendment of Sissach ash at a rate of 5% (w/w) to all tested biomasses (Figure 3B). All other ashes, when added to softwood, increased *y*_*N*_ by 22–120% compared to pyrolysis of nonamended softwood. An increase in *y*_*N*_ is linked to a lower amount of N being released to the gas phase, which could reduce the risk of NO_*x*_ formation during pyrogas combustion, a gas that is strictly regulated under national emission control.^34^ An AAEM-induced fixation of N in biochar, inhibiting the formation of NO_*x*_ precursors such as NH_3_ and HCN during pyrolysis, was observed in previous studies.^35,36^ Despite this, the differences in effects on *y*_*N*_ through the Sissach ash compared to all other ashes were not correlated to the ash properties, such as AAEM content, investigated in the present study.

#### 3.1.4 Reduction of Cr­(VI) during Pyrolysis

Due to elevated Cr­(VI) contents, four out of the six ash/coke samples would not have been allowed for soil application in Europe (Table S4 and Chapter 2.1 in the SI).^37–39^ However, none of the biochars produced from softwood amended with 0%, 10% or 20% of the highly Cr­(VI)-contaminated Brislach ash contained Cr­(VI) above the quantification limit of 0.1 mg kg^–1^. If Cr­(VI) were inert during feedstock preparation and biochar production, biochar would contain 6 to 8 mg Cr­(VI) kg^–1^, with a 10% and 20% ash amendment, respectively, which is well above the quantification limit. Recovery rates of total Cr in ash-amended biochars were 93% and 152% for 10% and 20% ash amendment, respectively; i.e., Cr volatilization during pyrolysis at 500 °C can be excluded. Recovery rates >100% can be explained by abrasion from the steel reactor, which does not interfere with Cr­(VI) quantification. If elevated Cr­(VI) contents prevented the use of ash as fertilizer, the AAEM nutrients it contains, in particular K, would be lost when disposed of as waste. Pyrolysis that eliminates Cr­(VI) could enable AAEM nutrient recycling, as such treated ashes could be applied to soil embedded in biochar.

The mechanism of Cr­(VI) reduction during biochar production could not be clearly elucidated, but several hypotheses can be formulated. First, Cr­(VI) might be reduced to Cr­(III) due to the reductive conditions in the pyrolysis reactor, i.e., the oxygen contained in chromate or dichromate (CrO_4_^2–^ and Cr_2_O_7_^2–^) is released during pyrolysis and used up as an oxidizing agent. Second, the contact of Cr­(VI) with the lignocellulose matrix during pelleting might reduce it to Cr­(III), as described for the “fixation process” when wood is impregnated with the Cr­(VI)-containing preservative chromium–copper arsenate (CCA).^40^ Third, the moistening of the wood and ash mixture prior to pelleting to a water content of 25% might lead to Cr­(VI) reduction, as is observed after moistening and storage of wood ash for several weeks, where water limits oxygen transport within the solid particles, leading to reduction to Cr­(III).^13,14,41^ The relevance of these potential mechanisms would need further investigation.

### 3.2 Impact of Ash Amendment on Biochar Production at Industrial Scale

#### 3.2.1 Biochar Yield

During pyrolysis at industrial scale, amending landscaping wood with 4% Gruyère_large ash increased *y*_*M*_ by 30% (*p* < 0.001), *y*_daf_ by 11% (*p* < 0.0021), and *y*_*C*_ by 7% (*p* < 0.0021) compared to the nonamended feedstock (Table 1). This demonstrates the potential of using ash as an additive for woody feedstocks in industrial biochar production, even when only adding loose ash intermittently to the wood in the biomass feed container, without moistening, pelleting, or extensive homogenization. The increase in *y*_daf_ observed at industrial scale was at the lower end of the range seen in experimental-scale trials (13%–38%, Figure 2), likely due to the lower ash dosage (4% vs 5%), a relatively high intrinsic ash content in the landscaping wood (11%), and the absence of feedstock pelleting. Simply adding ash to wood in the biomass feed container without pelleting is a more practical and cost-effective approach for operators.

Converted to the annual production of this specific pyrolysis plant (2200 t a^–1^ biomass throughput with a carbon content of 50%, dry matter), the yearly produced amount of biochar could increase from 810 to 1050 t (based on observed *y*_*M*_, Table 1) and the C-sink potential could increase from 2154 t CO_2_ equivalents (CO_2_e) to 2293 t CO_2_e (based on observed *y*_*C*_ and a conversion factor of 3.67 CO_2_e per amount of C). With a market value of € 800 t^–1^ of biochar and € 100–250 t^–1^ of sequestered CO_2_e,^42^ this corresponds to an increase in revenue of € 194,000 due to a higher amount of physical biochar and € 13,900–34,750 for additional C-sink certificates. Thus, with little effort for plant operators, cycles of AAEM nutrients can be closed, additional C can be sequestered, and pyrolysis plants can become more economic. However, the effect of ash addition on biochar degradation when applied to soil has not been investigated yet. The increased H/C_org_ ratio may indicate increased degradation.^43^

#### 3.2.2 Biochar Properties

The ash-amended biochar produced had a similar H/C_org_ molar ratio as the nonamended biochar (Table 1), in contrast to the increase observed at pilot-plant scale. The ash amendment increased *y*_*N*_ in biochar by 65%, similar to the observation on pilot-plant scale with the Gruyère ash sample (44% increase in *y*_*N*_ at pilot scale); thus, lower amounts of N were released to the gas phase under ash amendment.

The PTE content of biochars increased compared to the nonamended biochar (Table S7), as was expected due to their nonvolatile character during pyrolysis.^44^ Still, the ash-amended biochar met the limit values for the EBC AgroOrganic certification for contents of PTE (Table S7).^33^ The sum of the 16 polycyclic aromatic hydrocarbons prioritized by the Environmental Protection Agency (16 EPA PAH) remained largely unchanged by the ash amendment and remained within the EBC guideline (Table S8), in line with the literature.^9^ Also, it is well established that contamination of biochars obtained from slow-pyrolysis with organic compounds is a matter of process control, i.e., separation of pyrolysis gases and biochar at sufficiently high temperature to avoid contaminant condensation onto biochar.^45–47^

### 3.3 Legislative Status Quo for Use of Wood Ash as an Additive in European Biochar Production

The application of wood ash as an additive in biomass pyrolysis is permitted according to the EBC to date,^33^ as long as the limit values of the resulting biochar are met, which are related to soil protection and fertilizer guidelines, e.g., in Germany, Switzerland, and the EU.^48–50^ According to Regulation 2019/1009 of the European Union (EU, Fertiliser Product Regulation) under component material category (CMC) 14, the use of additives of up to 25% (w/w) to feedstock materials is permitted in pyrolysis, provided that they “improve the process performance or the environmental performance of the pyrolysis or gasification process”.^38^ Such additives, however, are not allowed if they are considered wastes according to Directive 2008/98EC (Article 3, point 1).^51^ In this context, ashes are often considered waste, as they are a byproduct of wood combustion, where the primary product is heat, and the ash is usually discarded. Therefore, the legal basis for the use of ash in biochar production for soil application still requires careful examination on a case-by-case basis, whereas the application of AAEM salts is straightforward.^7^ Also, we are not aware of any comparable restrictions on the use of ash-amended biochar in (construction) materials, although it does not contribute to nutrient recycling.

### 3.4 Practical Implications and Future Research

Ash amendment in biochar production is a useful tool for increasing the conversion rate of biomass-C into biochar-C, as demonstrated in the present study for various ash and biomass types, both at a pilot-plant and industrial scale. While we could not provide mechanistic insights beyond the literature, ash amendment consistently increased *y*_daf_ and *y*_C_ for natural woody feedstocks, with the positive effect being independent of their intrinsic ash content (tested with up to 11% intrinsic ash content in landscaping wood). In contrast, we found no evidence that ash addition can enhance yields in gramineous biomass. More research is needed to understand the mechanisms by which different ashes influence pyrolysis. For the time being, AAEM solubility may serve as a proxy for the effectiveness of ash as an additive in biochar production. Ash amendment on industrial pyrolysis plants can be implemented directly without pelletizing biomass and ash, which would enhance the carbon sequestration potential of industrial biochar production and enable ash-derived nutrient recycling. Furthermore, pyrolysis should be discussed and researched as a viable tool for treating Cr­(VI)-contaminated biomass streams. Yet, when dealing with Cr­(VI)-contaminated ashes as pyrolysis additives, workplace safety has to be assured by using skin and respiratory protection, and ash dosage must be carried out in such a way that no ash dust is released to the environment.

Beyond PyCCS, BECCS also stands out as a crucial negative emission technology, often relying on wood combustion. Our industrial-scale demonstrations now clearly show that these two technologies can be effectively integrated through the strategic use of ash in pyrolysis. This approach proves to be beneficial even with biomasses already high in ash, like bark or landscaping wood, which are only partially suitable for combustion. To facilitate the broader adoption of this combined strategy, the EU must integrate regulations to simplify the use of ash additives in biochar production.

## Supplementary Material



## Notes

Materials and methods, ash and biomass properties, correlation analysis results, experiments of influence of feedstock pelleting on biochar yield, and properties of biochars produced on industrial scale (PDF)
