# Supplementary material for: Increasing Biochar Yield in Pyrolysis by Adding Ash
Source: Environ Sci Technol. 2026 Aug 4;60(32):22314–22. doi: 10.1021/acs.est.5c14739 (PMC13492290; doi:10.1021/acs.est.5c14739)
Supplement: Supplementary file 1 [file es5c14739_si_001.pdf]

Increasing biochar yield in pyrolysis by adding ash

Jannis Grafmüller[1,2,3,*], Michael Tobler[4], Philipp Vögelin[5], Thomas D. Bucheli[3], Hans-Peter Schmidt[1], Nikolas Hagemann[1,3]

[1]: Ithaka Institut, Arbaz, Switzerland and Goldbach, Germany

[2]: Institute of Sustainable Energy Systems (INES), Offenburg University, Offenburg, Germany

[3]: Environmental Analytics, Agroscope, Zurich, Switzerland

[4]: Holz & Forst Consulting GmbH, 4102 Binningen, Switzerland

[5]: Industrielle Werke Basel (IWB); Margarethenstrasse 40; 4002 Basel, Switzerland

*Corresponding author: grafmueller@ithaka-institut.org

Number of pages: 27

Number of figures: 7

Number of tables: 8

Supporting Information

1. Supplemental description of Materials and Methods

1.1. Additional information on wood ashes

Table S1: *Origins of the wood ashes used for the pyrolysis experiments and their particle size fractions.*

Ash description	Ash Type	Biomass feedstock	Mass Fraction >2mm [%]	Mass fraction <2mm [%]
Sissach	Bottom ash	Natural forest wood (hard and softwood)	17%	83%
Brislach	Bottom ash	Natural forest wood (hard and softwood)	7%	93%
Möhlin	Bottom ash	Natural forest wood (hard and softwood)	6%	94%
Gruyère	Bottom ash	Sawmill residual wood without bark (softwood)	41%	59%
Gruyère_large[a]	Bottom ash	Sawmill residual wood without bark (softwood)	n.d.	n.d.
Zeglingen	Wood gasifier coke	Sawmill residual wood with bark (softwood)	27%	73%

[a]: Gruyère_large was used for industrial pyrolysis

1.2. Pelletized feedstocks for pyrolysis at pilot-plant scale

Table S2*: All biomass and ash mixtures used for pelleting for the pyrolysis experiments at pilot-plant scale. The analyses of the ashes and biomasses can be found in Tables S4 and S5, respectively.*

Biomass ID	Biomass (mixture)	Ash amendment to feedstock [%] (w/w, dry)	Ash type
Effect of ash amendment under variation of intrinsic biomass ash content			
1	100% Softwood	0	n.a.
2	75% Softwood	0	n.a.
	25% Grass		n.a.
3	50% Softwood	0	n.a.
	50% Grass		
4	25% Softwood	0	n.a.
	75% Grass		
5	100% Grass	0	n.a.
6	100% Softwood	5	Sissach
7	75% Softwood	5	Sissach
	25% Grass		
8	50% Softwood	5	Sissach
	50% Grass		
9	25% Softwood	5	Sissach
	75% Grass		
10	100% Grass	5	Sissach
Effect of ash amendment on pyrolysis of different feedstocks			
1	Softwood	0	n.a.
11	Forest Wood	0	n.a.
12	Waste Timber	0	n.a.
13	Straw	0	n.a.
14	Bark	0	n.a.
6	Softwood	5	Sissach
15	Forest Wood	5	Sissach
16	Waste Timber	5	Sissach
17	Straw	5	Sissach
18	Bark	5	Sissach
Effect of ash amendment dependent on ash type			
1	Softwood	0	n.a.
6	Softwood	5	Sissach
19	Softwood	5	Gruyère
20	Softwood	5	Brislach
21	Softwood	5	Möhlin
22	Softwood	5	Zeglingen
Effect of Pyrolysis on Cr(VI)			
1	Softwood	0	n.a.
23	Softwood	10	Brislach
24	Softwood	20	Brislach

1.3. Reactor scheme of industrial pyrolysis plant

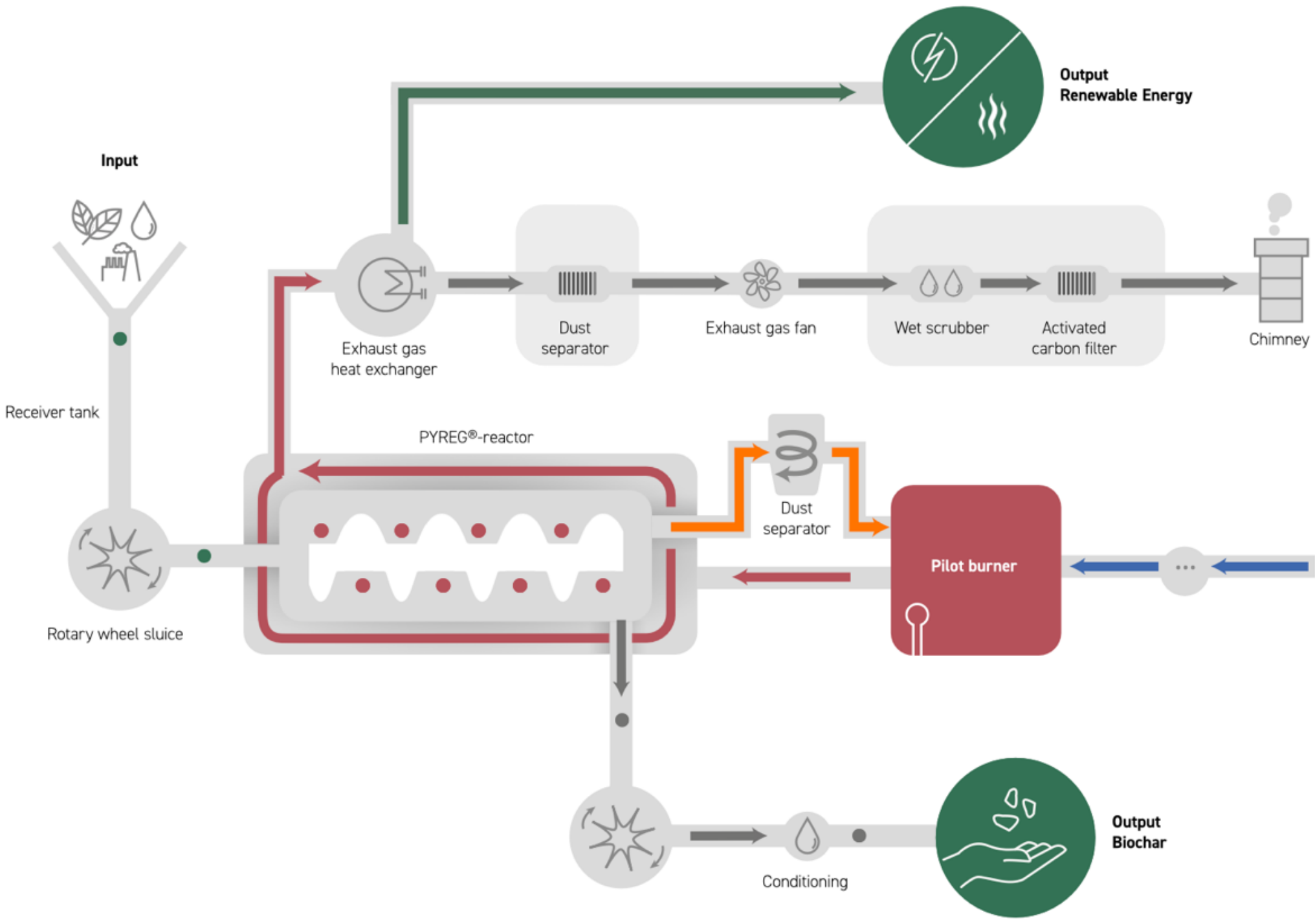

Figure S1: *Process scheme of industrial pyrolysis plant PX1500 (Pyreg GmbH, Dörth, Germany). Scheme was retrieved from the manufacturer's website, where further information can be found: https://pyreg.com/our-technology/.*

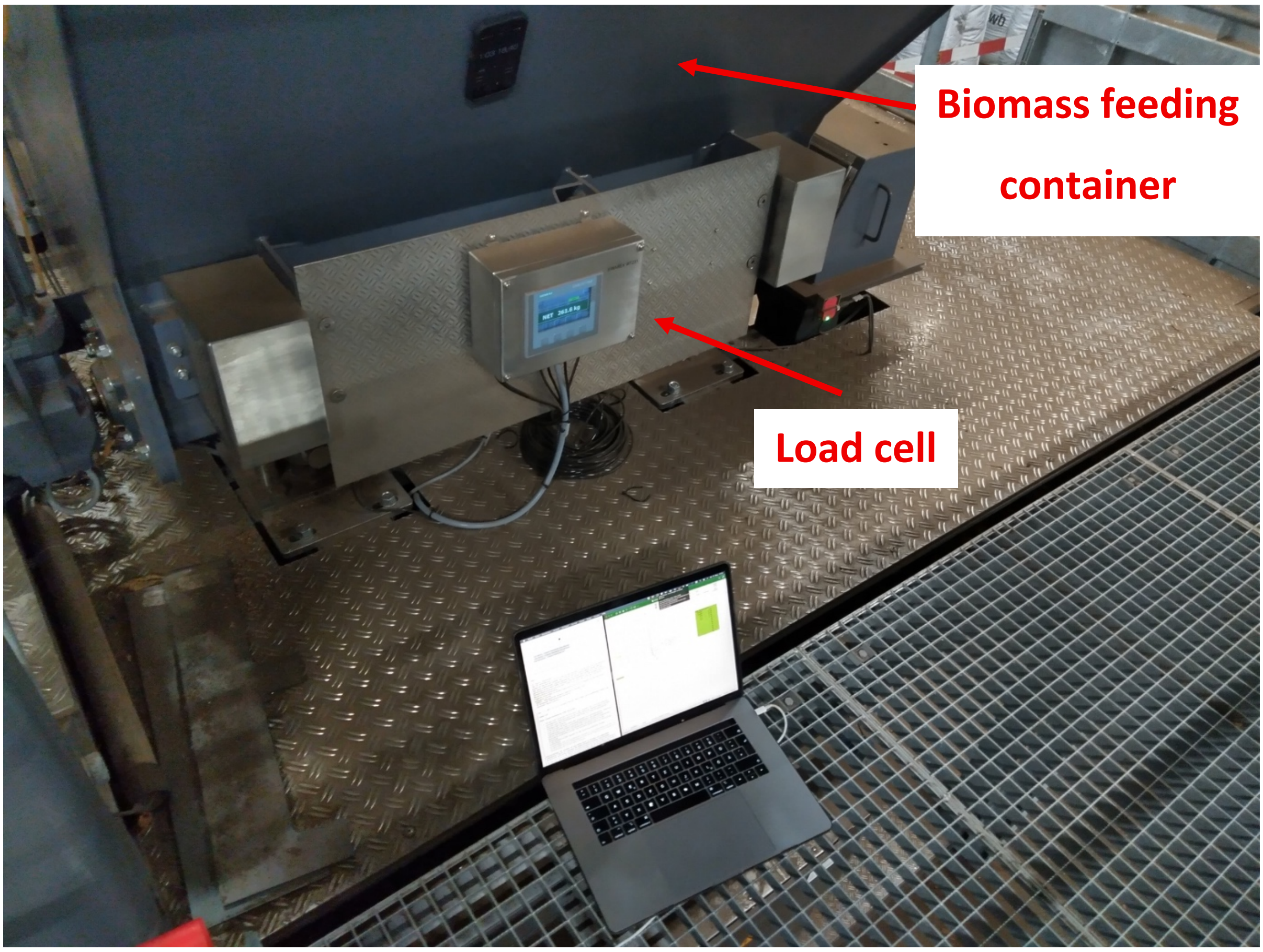

Figure S2: *Load cell installed at the biomass feeding container. The load cell was recorded during the 15-minute time intervals during which the container was not filled but only emptied into the reactor. This way, a biomass mass flow into the reactor was obtained as an average value during the whole experiment.*

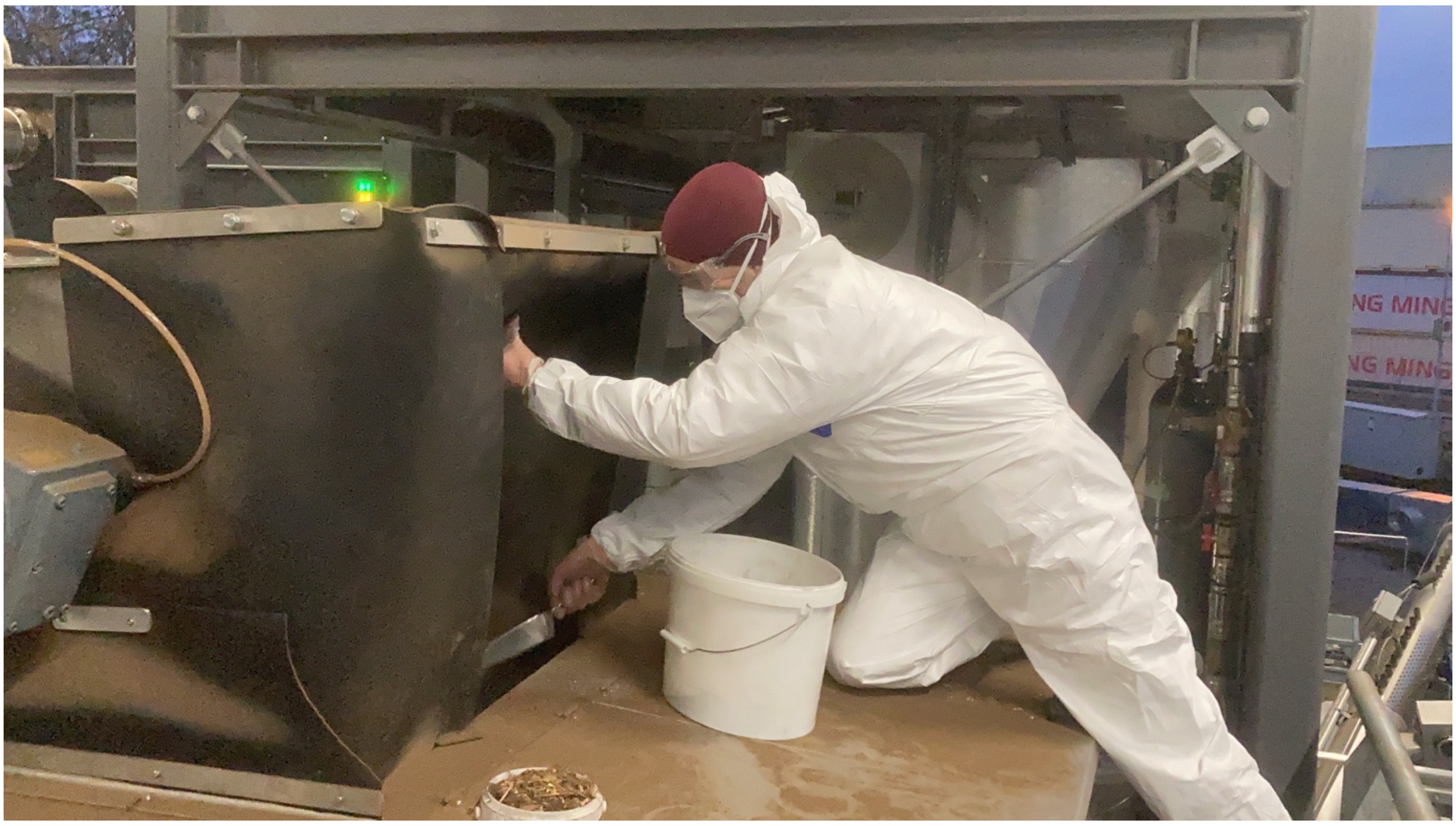

Figure S3: *Manual dosing of wood ash to the biomass feeding container installed on top of the pyrolysis reactors. The ash was added to the container during the short time interval (1 minute) during which the bunker was filled with wood, which happened every 15 minutes.*

1.4. Ash, biomass and biochar analysis

Table S3: *Ash, biomass and biochar analysis performed by Eurofins Umwelt Ost GmbH (Bobritzsch-Hilbersdorf, Germany) in accordance with the respective regulations.*

Sample matrix	Parameter	References
Ash	pH	DIN ISO 10390[1]
Ash	Ash content (550 °C)	DIN EN 15169[2]
Ash	Main Elements	DIN EN ISO 17294-2[3]
Ash	Trace Elements	DIN EN ISO 17294-2<br>DIN EN ISO 12846[4]
Ash, Biomass	Total Carbon	DIN EN ISO 16948[5]
Biomass	Ash content (550 °C)	DIN EN ISO 18122[6]
Biomass	Main and trace elements	DIN EN ISO 11885[3]<br>DIN EN ISO 17294-2[3]08.07.26 11:26:00
Biomass	Cellulose, Hemicellulose Lignin content	VDLUFA Method 6.5.3, volume III , 2012[7]
Biochar	Ash content (550 °C)	European Biochar Certificate[8]
Biochar	Carbon, Hydrogen, Nitrogen, Sulfur content	European Biochar Certificate[8]
Biochar	Polycyclic Aromatic Hydrocarbons (16 EPA PAH)	European Biochar Certificate[8]
Biochar, Ash	Hexavalent chromium	DIN EN 16318[9]

1.5. Quantification of biochar mass yields

For the measurement of feedstock mass flow ($f_{feedstock}$, equation (1)), PYREKA was operated at room temperature to determine the mass of feedstock being transported through the reactor in a given time t ($M_{feedstock,t}$). Mass yield (y_M) of biochar was calculated as follows in equation (2):

$$f_{feedstock} = \frac{M_{feedstock,t}}{t} \qquad (1)$$

$$y_M = \frac{M_{biochar,t}}{t} \cdot \frac{1}{f_{feedstock}} \cdot 100\% \qquad (2)$$

where $M_{biochar,t}$ is the amount of biochar produced over the time period t. An average value for $f_{feedstock}$ was obtained by four replicate measurements recorded with $t \geq 10$ min. Mass of biochars during pyrolysis was determined without replicates with a one-time sampling interval of three residence times (RT), i.e., $t = 30$ min. Further, $f_{feedstock}$ was corrected for its dry matter content, which was determined in duplicates at 105 °C. The mass of biochar was measured instantly after production, which allowed assuming a dry matter content of 100%.

1.6. Quantification of dry and ash-free yield (y_{daf}) and carbon yield (y_C)

For each biochar sample, y_{daf} and y_C was calculated according to equation (3) and (4):

$$y_{daf} = y_M \cdot \frac{(1 - a_{BC})}{(1 - a_f)} \quad (3)$$

$$y_C = y_M \cdot \frac{c_{C,BC}}{x_{a,f} \cdot c_{C,a} + \sum_{n=0}^{i} x_{BM,i} \cdot c_{C,BM}} \quad (4)$$

where y_M is the dry biochar yield according to equation (2), a_{BC} and a_f are the ash contents of biochar and feedstock, respectively, $c_{C,BC}$, $c_{C,BM}$ and $c_{C,a}$ represent the total C content in the biochar, biomass and ash additive, respectively, $x_{a,f}$ and $x_{BM,i}$ represent the mass fraction of ash additive and each individual biomass in the pelleted feedstock. For the ash additive, $c_{C,a}$ was assumed to equal the loss on ignition of the sample at 850 °C. For the Zeglingen sample, the C content was separately quantified by elemental analysis.

1.7. Quantification of nitrogen yield increase

For ash addition in industrial pyrolysis, the change in nitrogen yield (y_N) was calculated based on nitrogen content (x_N) and mass yield (y_M) of pure (p) and ash-amended (a) biochar:

$$\Delta y_N = \frac{x_{N,a} \cdot y_{M,a}}{x_{N,p} \cdot y_{M,p}} \cdot 100\% - 100\% \quad (5)$$

1.8. Quantification of deviation of biochar yield on pilot-plant scale according to error propagation law

1.8.1. Biochar mass yield y_M

$$\Delta\, y_M = \sqrt{\left(\frac{\delta y_M}{\delta M_{biochar,t}} \cdot \Delta M_{biochar,t}\right)^2 + \left(\frac{\delta y_M}{\delta f_{feedstock}} \cdot \Delta f_{feedstock}\right)^2 + \left(\frac{\delta y_M}{\delta t} \cdot \Delta t\right)^2} \quad (6)$$

With $\Delta M_{biochar,t} = \Delta t = 0$ (not measured in replicates):

$$\Delta\, y_M = \sqrt{\left(\frac{\delta y_M}{\delta f_{feedstock}} \cdot \Delta f_{feedstock}\right)^2} = \sqrt{\left(-\frac{M_{biochar,t}}{t} \cdot \frac{1}{f_{feedstock}{}^2} \cdot \Delta f_{feedstock} \cdot 100\%\right)^2} \quad (7)$$

With $\Delta f_{feedstock}$ being the standard error of the feedstock mass flow.

1.8.2. Dry and ash-free biochar yield y_{daf}

$$\Delta\, y_{daf} = \sqrt{\left(\frac{\delta y_{daf}}{\delta y_M} \cdot \Delta y_M\right)^2 + \left(\frac{\delta y_{daf}}{\delta a_{BC}} \cdot \Delta a_{BC}\right)^2 + \left(\frac{\delta y_M}{\delta a_f} \cdot \Delta a_f\right)^2} \quad (8)$$

With $\Delta a_{BC} = \Delta a_f = 0$ (no replicated ash content analysis) and Δy_M from equation 7:

$$\Delta\, y_{daf} = \sqrt{\left(\frac{\delta y_{daf}}{\delta y_M} \cdot \Delta y_M\right)^2} = \sqrt{\left(\frac{(1 - a_{BC})}{(1 - a_f)} \cdot \Delta y_M\right)^2} \quad (9)$$

1.8.3. Carbon yield y_C

With $c_{C,BC} = x_{a,f} = c_{C,a} = x_{BM,i} \cdot c_{C,BM} = 0$ (no replicated C analysis, no deviation in x_{BM} and $x_{a,f}$ quantified), the deviation of y_C is calculated as follows:

$$\Delta y_C = \sqrt{\left(\frac{\delta y_C}{\delta y_M} \cdot \Delta y_M\right)^2} = \sqrt{\left(\frac{c_{C,BC}}{x_{a,f} \cdot c_{C,a} + \sum_{n=0}^{i} x_{BM,i} \cdot c_{C,BM}} \cdot \Delta y_M\right)^2} \tag{10}$$

1.9. Quantification of increases in y_{daf} and y_C

The increase in y_{daf} and y_C was calculated based on yields of ash-amended biochar (index a) related to those of the pure, non-amended biochar (index p):

$$y_{daf,In.} = \frac{y_{daf,a}}{y_{daf,p}} \cdot 100\% - 100\% \tag{11}$$

$$y_{C,In.} = \frac{y_{C,a}}{y_{C,p}} \cdot 100\% - 100\% \tag{12}$$

1.9.1. Deviation of $y_{daf,In.}$

With $y_{daf,a}$ and $y_{daf,p}$ being the y_{daf} of biochar production from a similar biomass with (index a) or without (index p) an ash amendment and $\Delta y_{daf,a}$ as well as $\Delta y_{daf,p}$ being the deviations of y_{daf} according to equation 9:

$$\Delta y_{daf,In.} = \sqrt{\left(\frac{\delta y_{daf,In}}{\delta y_{daf,a}} \cdot \Delta y_{daf,a}\right)^2 + \left(\frac{\delta y_{daf,In}}{\delta y_{daf,p}} \cdot \Delta y_{daf,p}\right)^2}$$

$$= \sqrt{\left(\frac{1}{y_{daf,p}} \cdot 100\% \cdot \Delta y_{daf,a}\right)^2 + \left(-\frac{y_{daf,a}}{{y_{daf,p}}^2} \cdot 100\% \cdot \Delta y_{daf,p}\right)^2} \tag{13}$$

1.9.2. Deviation of $y_{C,In.}$

With $y_{C,a}$ and $y_{C,p}$ being the y_C of biochar production from a similar biomass with (index a) or without (index p) an ash amendment and $\Delta y_{C,a}$ as well as $\Delta y_{C,p}$ being the deviations of y_C according to equation 10:

$$\Delta\, y_{C,In.} = \sqrt{\left(\frac{\delta y_{C,In}}{\delta y_{C,a}} \cdot \Delta y_{C,a}\right)^2 + \left(\frac{\delta y_{C,In}}{\delta y_{C,p}} \cdot \Delta y_{C,p}\right)^2}$$

$$= \sqrt{\left(\frac{1}{y_{C,p}} \cdot 100\% \cdot \Delta y_{C,a}\right)^2 + \left(-\frac{y_{C,a}}{{y_{C,p}}^2} \cdot 100\% \cdot \Delta y_{C,p}\right)^2} \qquad (14)$$

2. Supporting Results and Discussion

2.1. Properties of ashes and suitability for direct soil application

All ash samples had an ash content close to 100%, except for the Zeglingen sample, which was an ash-rich gasifier coke and had an ash content of 70% (Table S4) and a 25% C content. Among AAEM, Ca exhibited the highest amounts in the ashes, followed by K, Mg, and Na (Table S4). The total AAEM content was lowest in the Zeglingen and Sissach samples (333-387 g kg^{-1}) and highest in the Brislach sample (505 g kg^{-1}, Table S4). Overall, the composition of the ashes was well within the range reported in the literature.[10,11]

According to XRD analysis of the ashes, a large fraction of AAEM (42-57%, Figure S4b) was present in amorphous structures. In the crystalline phase, AAEM were predominantly present in form of carbonates, oxides, hydroxides, and silicates (Figure S4a and b). AAEM were present to a negligible extent as phosphates and sulphates (Figure S4b). The Zeglingen sample had a significantly higher fraction of AAEM bound as $CaCO_3$ compared to the other ashes. The Sissach sample had the highest share of AAEM bound as Ca_2SiO_4 (Figure S4b), a mineral that was found in all ashes except the Zeglingen sample. The Zeglingen sample was further the only ash with a relevant AAEM allocation in $K_2Ca_2(CO_3)_3$. The XRD measurements recovered 43-67% of total AAEM content quantified by wet chemistry analysis, which matches the finding that 42-57% (w/w) of the ash samples were of amorphous character and thus not covered by XRD. Based on the above findings, the ashes comply with the mineral fingerprint of wood ashes usually obtained in a temperature range of 700-800 °C.[12] Concentrations for cristobalite remained below the detection limit of 0.2 wt% in all ash samples.

For all ashes, water-solubility of K was highest compared to the other AAEM, both in absolute and relative amounts, contributing up to 100% to the soluble fraction of AAEM (Table S5). The Sissach and Gruyère samples shared the lowest solubility of total K (20-34%, respectively) and total AAEM, while it was highest for the Zeglingen sample (81%, Table S5), matching with the high content of K_2SO_4 and $K_2Ca_2(CO_3)_3$ in the Zeglingen sample. Water solubility of K was well related to the K content determined by XRD present in the crystalline phase in the samples (R^2=0.78, Figure S5), indicating that K contained in XRD-recovered crystalline structures in ashes and gasifier cokes might exhibit higher solubility compared to K in amorphous structures.

Copper, zinc, chromium, and nickel were the most relevant quantified potentially toxic elements (PTE) in the ashes (Table S4). Additionally, the Sissach sample had a high Pb content compared to the other ashes, since the latter were intendedly selected from burning chambers using wood from forests that were at significant distance from busy roads. Due to its overall low concentrations of PTE, the Gruyère ash was selected to be used for the scale-up to industrial pyrolysis, as PTEs accumulate in biochar. The second sample of the Gruyère ash (Gruyère_large), however, had higher Cu and Cr contents, highlighting the variability in composition of wood ashes over time, even within the same combustion plant using the same input biomass (Table S4). The Brislach and Möhlin samples were characterized by the highest Cr(VI) contents (Table S4).

The use of ash in agriculture is subject to legal framework requirements in the European Union (EU), where wood ash is categorized in the compound material category (CMC) 13 (Thermal Oxidation Materials or Derivates) under Product Function Category (PFC) 1 (Inorganic Macronutrient Fertiliser) in the EU Fertilizing Product Regulation.[15] There are also country-specific regulations for agricultural soil application of wood ash, e.g., in Germany (BGH) and Austria (BMLFUW).[16,17] All guidelines set a limit of 2 mg Cr(VI) kg^{-1} (Table S4), to which only Sissach and Zeglingen ashes were compliant. Still, due to high PTE content in Sissach, only the Zeglingen ash would have been allowed for soil application (Table S4). All ashes and the gasifier coke that were used in this study are currently disposed of by the producers (landfill with special requirements), highlighting the necessity to develop recycling procedures to avoid losing plant nutrients from natural material cycles.

Table S4: *Elemental composition of wood ashes used for the pyrolysis experiments and limit values according to selected regulations. The Zeglingen sample was an ash-rich gasifier coke, all other samples were bottom ashes from biomass burning chambers. Phosphorus (P), calcium (Ca), magnesium (Mg), sodium (Na), potassium (K), lead (Pb), cadmium (Cd), copper (Cu), nickel (Ni), mercury (Hg), zinc (Zn), total chromium (Cr), hexavalent Cr (Cr^{VI}), arsenic (As), thallium (Tl). n.d.: not defined. The last three rows indicate compliance with various regulations.*[g]

	Ash Samples						Regulations		
	**Sissach**	**Brislach**	**Möhlin**	**Gruyère**[a]	**Gruyère_large**[a]	**Zeglingen**	**BGH**[b]	**BMLFUW A/B**[c]	**EU-FPR**[d]
**Ash** [%]	98.5	99.5	99.3	96.6	100.0	70.4	n.d.	n.d.	n.d.
**P** [g kg^{-1}]	14.4	11.8	12.9	17	13.9	5.6	n.d.	n.d.	n.d.
**Ca** [g kg^{-1}]	287.0	388.0	305.0	369.0	349.0	236.0	n.d.	n.d.	n.d.
**Mg** [g kg^{-1}]	19.7	37.9	33.9	18.3	24.0	12.2	n.d.	n.d.	n.d.
**Na** [g kg^{-1}]	2.4	0.6	1.9	2.1	2.4	0.2	n.d.	n.d.	n.d.
**K** [g kg^{-1}]	77.9	78.5	103.0	43.1	66.9	84.9	n.d.	n.d.	n.d.
**Sum of AAEM**[f] [g kg^{-1}]	387.0	505.0	443.8	432.5	442.3	333.3	n.d.	n.d.	n.d.
**Pb** [mg kg^{-1}]	183	3	8	5	7	5	150	100/200	120
**Cd** [mg kg^{-1}]	<0.2	1.3	0.2	0.6	<0.2	0.6	1.5	5/8	3
**Cu** [mg kg^{-1}]	166	89	225	104	230	88	900	200/250	600
**Ni** [mg kg^{-1}]	22	68	72	31	44	21	30	150/200	100
**Hg** [mg kg^{-1}]	<0.07	<0.07	<0.07	<0.07	<0.07	<0.07	1	n.d.	1
**Zn** [mg kg^{-1}]	96	107	82	157	49	592	5000	1200/1500	1500
**Cr** [mg kg^{-1}]	39	75	57	78	204	7	n.d.	150/250	400

**Cr(VI)** [mg kg^{-1}]	1.6	19.0	17.0	4.5	7.5	<0.1	2	2/2	2
**As** [mg kg^{-1}]	3.9	1.5	1.3	2.9	2.1	<0.8	40	20/20	40
**Tl** [mg kg^{-1}]	<0.2	<0.2	<0.2	<0.2	<0.2	<0.2	n.d.	n.d.	2
**BGH**	- (-)	- (-)	- (-)	- (+)	- (-)	+ (+)			
**BMLFUW**	-/+ (-/+)	- (+)	-/+(-/+)	- (+)	- (-/+)	+ (+)			
**EU-FPR**	- (-)	- (+)	- (+)	- (+)	- (+)	+ (+)			

[a]: Gruyère was used for pyrolysis on pilot-plant scale and Gruyère_large was used for the experiments on industrial scale.

[b]: Germany: Threshold values for wood ashes to be used as soil or compost amendment according to the German Federal Quality Association Wood Ash (BGH).[16]

[c]: Austria: Threshold values for the use of biomass ash on agricultural and forestry land according to the Austrian Ministry for Agriculture and Forestry, Environment and Water Management (BMLFUW) based on hydrofluoric acid digestion. Ashes that meet the limit values in category A are allowed for soil application without providing a soil analysis of the application site. Ashes with contents between threshold A and B are only allowed after providing a soil analysis of the application site and consideration of nutrient uptakes by plants.[17]

[d]: European Union: EU Fertilizer Product Regulation (EU 2019/1009, EU-FPR): combined limit values for component material category CMC 13 ("Thermal Oxidation Materials or Derivates") and product function category PFC 1(C)(I): Inorganic Macronutrient Fertilizer. There are additional limit values for vanadium and chlorine which are not considered here as their analysis was beyond the scope of this study.[15]

[f]: Sum of the alkali and alkaline earth metals (AAEM) Na, K, Mg, and Ca.

[g]: The compliance with regulations is evaluated when including or excluding (in brackets) Cr(VI) contents. Compliance is indicated with a '+' and non-compliance by '-'. For BMLFUW, compliance is evaluated against subcategories A or B, separated by '/'. If no '/' is indicated, evaluation refers to both subcategories.

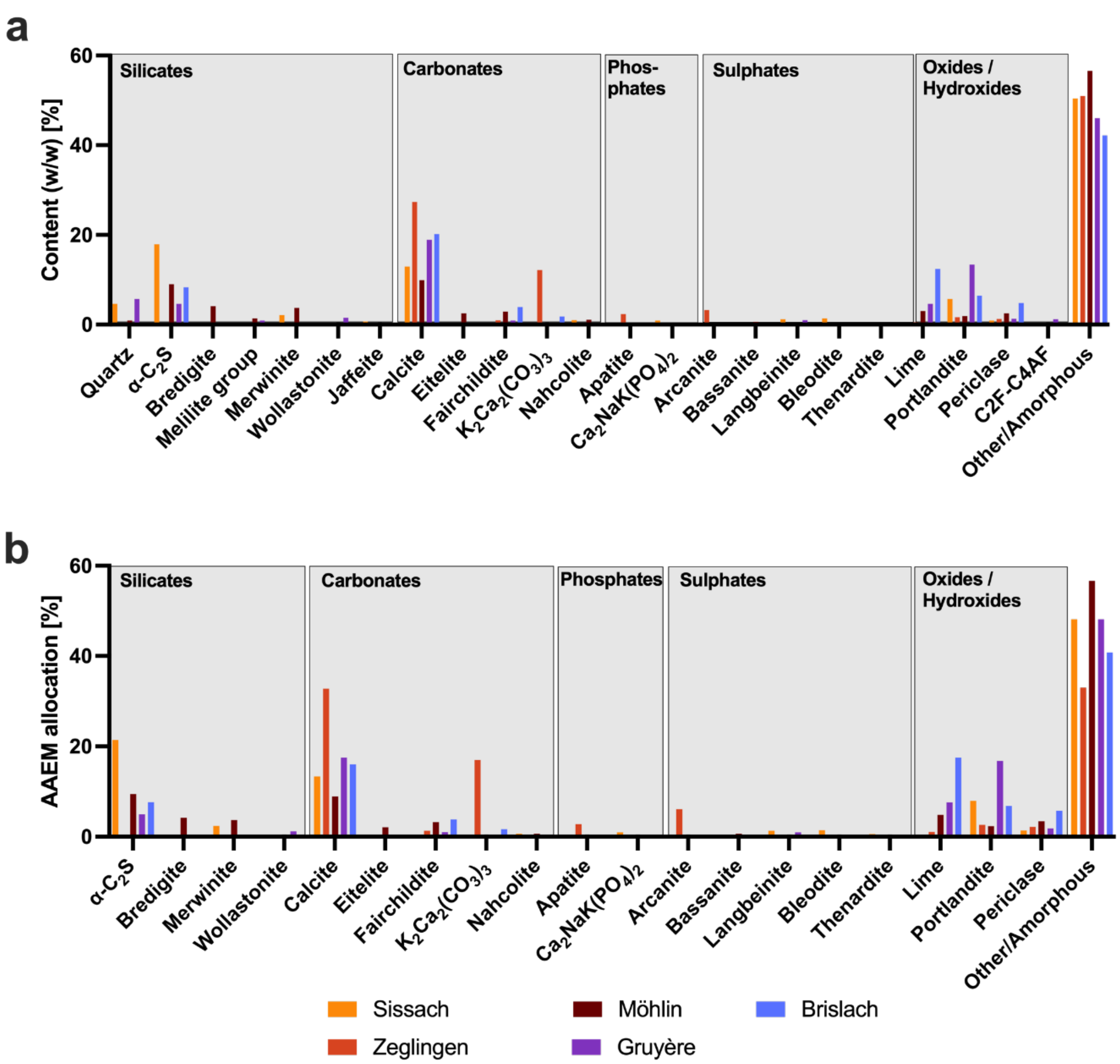

Figure S4: *Quantitative X-ray diffraction (XRD) analysis of the five wood ash samples expressed as total content (w/w) of the mineral in the sample (a). Panel b presents the fraction of alkali and alkaline earth metals (AAEM) quantified by wet chemistry analysis in a specific ash, i.e., potassium, sodium, magnesium and calcium, which is allocated in a specific mineral quantified by XRD. X-ray diffraction analysis of the ashes was performed after drying the samples at 40 °C and dry-milling with an internal standard prior to measurement in the diffractometer (D8 Advance equipped with an XE-T detector, irradiation with Cu-Kα-radiation, Bruker GmbH, Mannheim, Germany).*

Table S5: *Water soluble amount and fractions of alkali and alkaline earth metals (AAEM) calcium (Ca), magnesium (Mg), potassium (K) and sodium (Na) and their total sum from the different ash samples. Values are based on elution of the ashes (1+10, m+V) for 1 hour. Errors indicate the standard deviation of three replicates, where available.*

	water solubility [g kg^{-1}]					water solubility [%]				
**Sample**	**Ca**	**Mg**	**K**	**Na**	**∑AAEM**	**Ca**	**Mg**	**K**	**Na**	**∑AAEM**
**Sissach**	4.9	$9\cdot10^{-4}$	15	0.3	21	1.7	<0.01	20	13	5.0
**Zeglingen**	0.02 ± 0.0	$5\cdot10^{-4}$	69 ± 2	0.1 ± 0.0	69 ± 2	<0.1	<0.01	81 ± 3	57 ± 1	20.7 ± 0.6
**Möhlin**	2.2 ± 0.0	$2\cdot10^{-4}$	44 ± 1	0.4 ± 0.0	47 ± 1	0.7 ± 0.0	<0.01	43 ± 1	22 ± 1	10.5 ± 0.2
**Gruyère**	4.7 ± 0.1	$3\cdot10^{-4}$	15 ± 1	0.3 ± 0.0	20 ± 1	1.3 ± 0.0	<0.01	34 ± 1	13 ± 1	4.6 ± 0.1
**Brislach**	1.8 ± 0.1	$3\cdot10^{-4}$	50 ± 1	0.2 ± 0.0	52 ± 1	0.5 ± 0.0	<0.01	64 ± 1	35 ± 1	10.3 ± 0.2

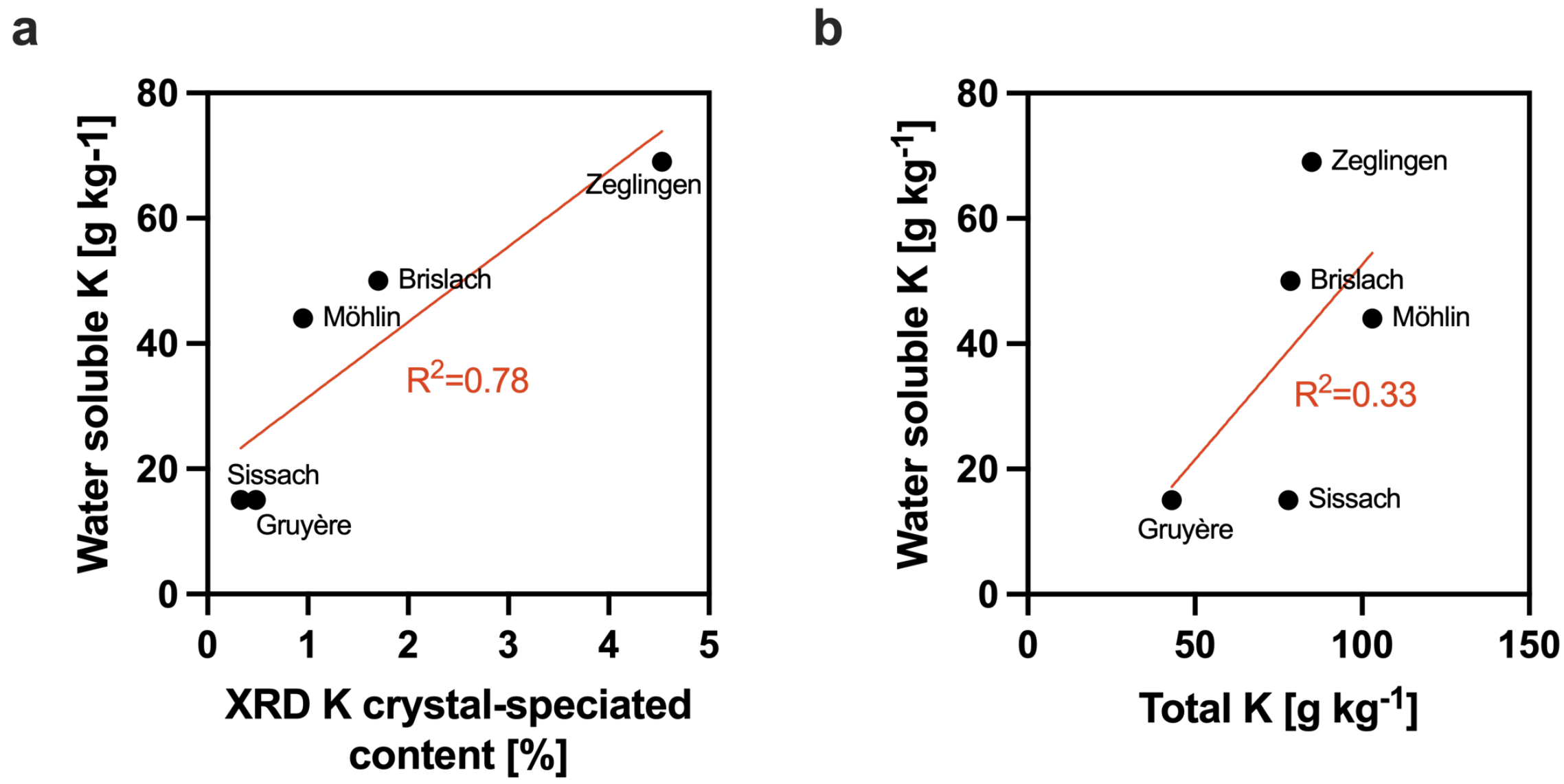

Figure S5: *Water soluble content of potassium (K) in the wood ash samples (Sissach, Gruyère,, Möhlin and Brislach) and the gasifier coke (Zeglingen) plotted over the K content in the ashes in crystalline structures according to X-ray diffraction analysis (panel a, in %, w/w) and plotted over the total K content in the samples determined by wet chemistry analysis (panel b). The* R^2 *values refer to linear regression through data points.*

2.2. Biomass properties

Elemental and macromolecular composition varied considerably between biomasses (Table S6). The landscaping wood had the highest ash content (11%) followed by bark, grass, and straw while the lowest of 0.4-1.1% were present in forest and softwood (Table S6). Carbon content was in a narrow range (44-50%), despite larger differences in ash content. Calcium was the AAEM most present in biomasses, followed by K, Mg and Na (Table S6). The sum of AAEM contents was highest for the straw and bark (~200 mg kg^{-1}), followed by the grass and waste timber sample (~90 mg kg^{-1}) and was lowest for the landscaping wood, forest wood and softwood (4.5-39 mg kg^{-1}, Table S6). Thus, despite ash contents in a similar range for some of the biomasses, contents of individual AAEM and their sum had variation coefficients between 90 and 170%.

Lignin contents were highest for bark and the landscaping wood (42-45% (w/w)) and in a medium range for the other wood-based feedstocks (17-26% (w/w), Table S6). Straw and grass samples had the lowest lignin content (7%). Cellulose contents were highest for softwood, forest wood, and waste timber (55-61%). All other feedstocks had cellulose contents between 36 and 41%. Grass and straw had the highest hemicellulose contents (27-32%), followed by softwood, forest wood, and waste timber (16-22%), while bark as well as landscaping wood had only 3.1% of hemicellulose.

The softwood sample was delivered bark-free, while forest wood was delivered and processed with bark. The landscaping wood contained a high proportion of branches, explaining its relatively high lignin content. Further, this sample might have been partly microbial digested during storage, explaining its lower C and relatively higher ash contents, the latter might also have come from foliage residues.

Table S6: *Contents of Carbon, ash, lignin, cellulose, hemicellulose, potassium (K), calcium (Ca), magnesium (Mg), phosphorus (P), and sulfur (S) in the biomasses used for the pyrolysis experiments (dry basis).*

**Parameter**	**Softwood**	**Grass**	**Forest Wood**	**Straw**	**Waste Timber**	**Bark**	**Landscaping Wood**[a]
**Carbon** [%]	50	47	50	44	49	49	48
**Ash** [%]	0.4	7.5	0.9	5.9	1.1	6.7	11
**Lignin** [%]	25	7	18	7	26	45	42
**Cellulose** [%]	55	36	61	40	55	39	41
**Hemicellulose** [%]	16	28	22	32	18	3	3
**K** [mg kg^{-1}]	1	23	10	160	55	55	6
**Ca** [mg kg^{-1}]	0.5	9	18	51	18	125	28
**Mg** [mg kg^{-1}]	1	21	2	3	3	13	3
**Na** [mg kg^{-1}]	2	37	1	2	5	4	2
**Sum of AAEM**[f] [mg kg^{-1}]	4.5	90	31	216	81	197	39
**P** [mg kg^{-1}]	1	25	1	4	1	17	3
**S** [mg kg^{-1}]	1	26	8	55	2	9	1
**Pb** [mg kg^{-1}]	<2	<2	<2	<2	12	3	<2
**Cd** [mg kg^{-1}]	<0.2	<0.2	<0.2	<0.2	0,2	<0.2	<0.2
**Cu** [mg kg^{-1}]	<1	4	2	11	21	8	6
**Ni** [mg kg^{-1}]	<1	4	1	2	1	8	4
**Hg** [mg kg^{-1}]	<0.05	<0.05	<0.05	<0.05	<0.05	<0.05	<0.05

**Zn** [mg kg^{-1}]	4	22	6	9	165	37	25
**Cr** [mg kg^{-1}]	<1	11	2	6	22	12	9
**As** [mg kg^{-1}]	<0.8	<0.8	<0.8	<0.8	13.9	<0.8	<0.8

[a]: Biomass used for pyrolysis at industrial scale

2.3. Further results on pyrolysis experiments

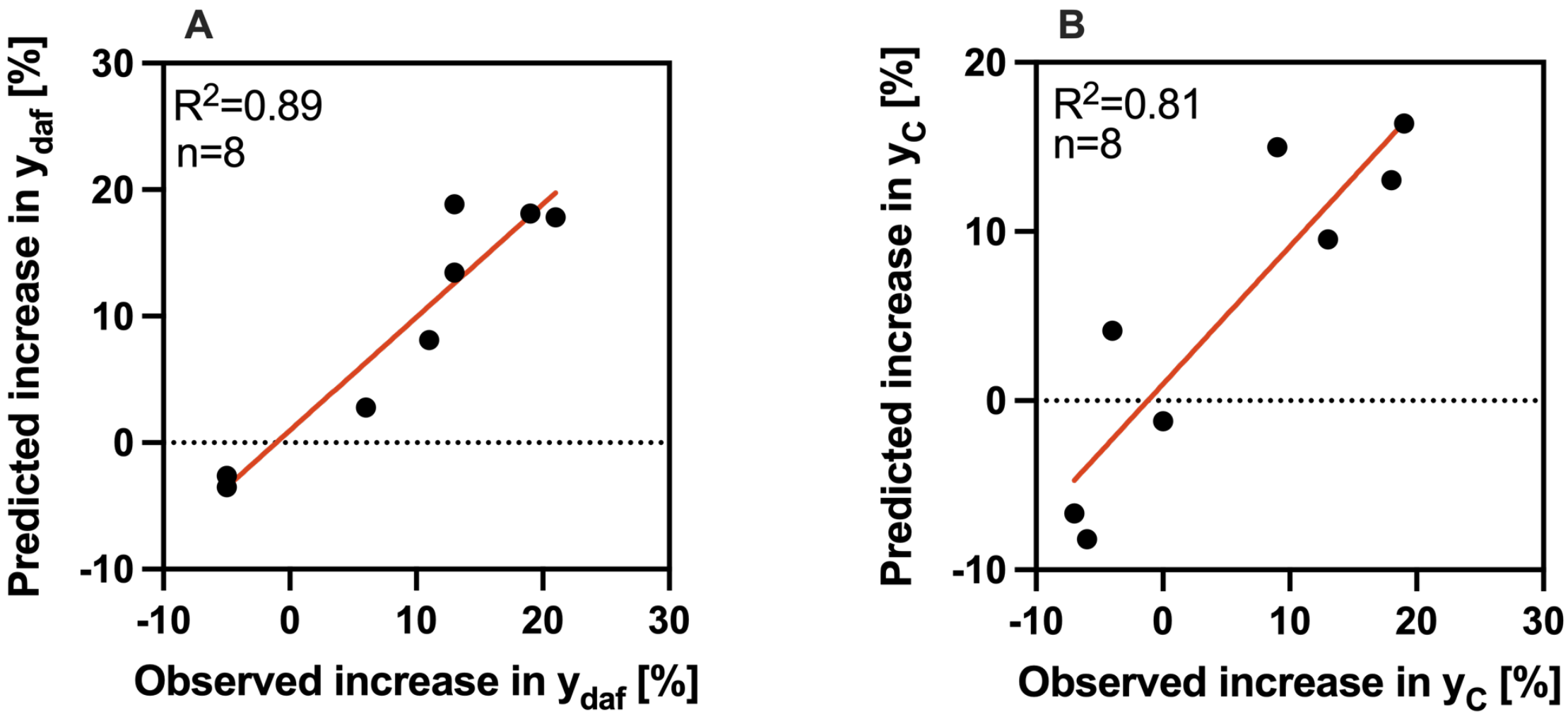

$$\Delta y_{daf} = -4.3 + 0.6 \cdot x_{Cellulose} - 0.8 \cdot x_{Hemicellulose}$$

$$\Delta y_{C} = -3.8 + 0.6 \cdot x_{Cellulose} - 0.9 \cdot x_{Hemicellulose}$$

Figure S6*: Predicted values of increase in dry and ash-free biochar yield (y_{daf}, a) or carbon yield (y_C, b) over observed increases in y_{daf} and y_C, respectively, in pyrolysis experiments using all biomasses, except waste timber, amended with 5% (w/w) of the Sissach ash performed at 500 °C with 10 minutes residence time. The predicted values were obtained from the presented multivariate models below the panels. The correlation coefficients correspond to the linear regression of predicted vs. observed values. $x_{Cellulose}$ and $x_{Hemicellulose}$ correspond to intrinsic contents of cellulose and hemicellulose in the biomasses, respectively. The other two input variables, i.e., intrinsic lignin and ash contents, had no significant impact on the model ($p>0.05$).*

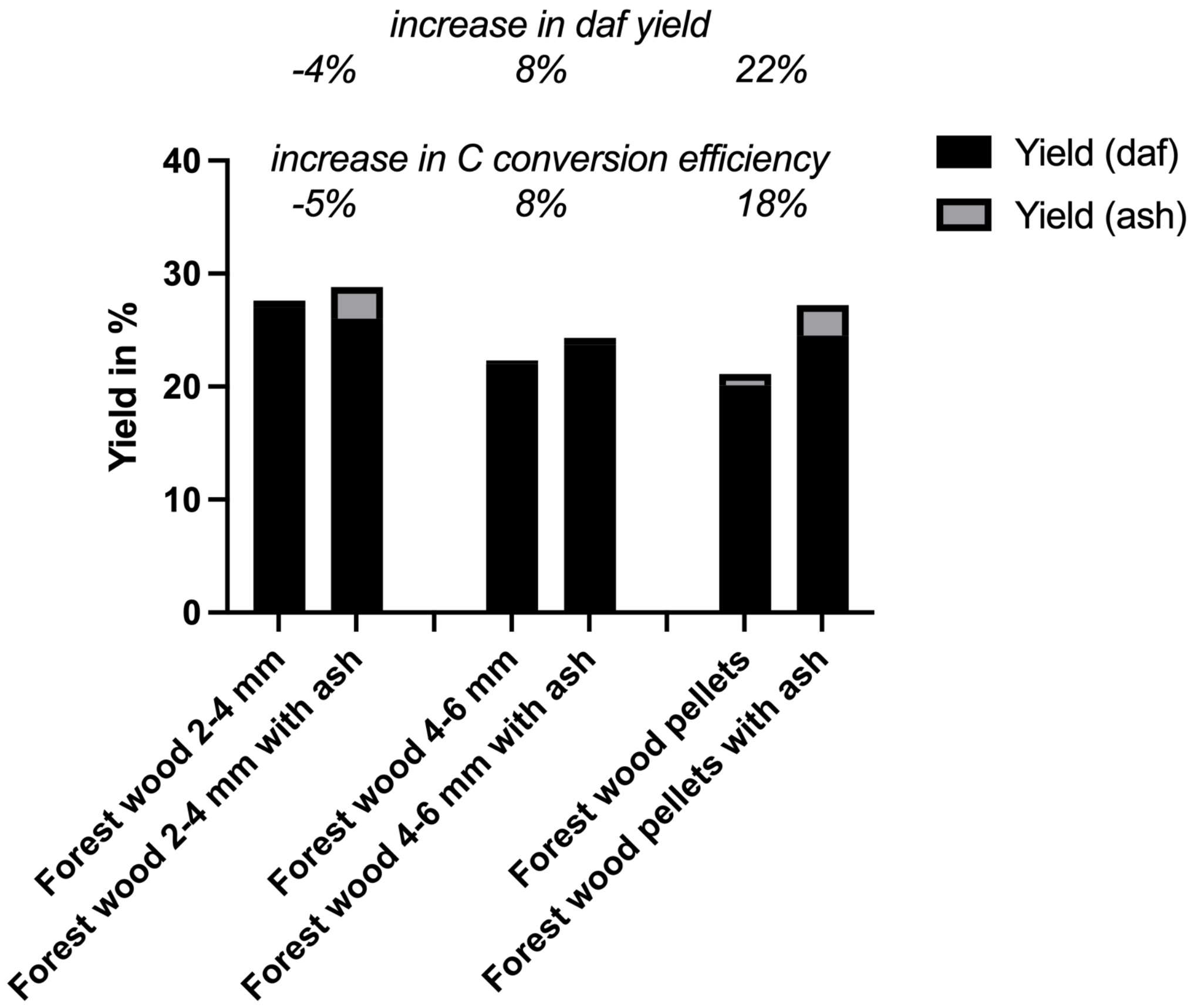

Figure S7: *Yield of biochar production (total and dry and ash free (daf)) of the sieved forest wood (2-4 mm and 4-6 mm) in loose mixture with the amended ash in comparison to the pelleted forest wood amended without or with ash.*

Table S7: *Potentially toxic elements biochars from the industrial pyrolysis experiment: arsenic (As), lead (Pb), cadmium (Cd), copper (Cu), nickel (Ni), quicksilver (Hg), zinc (Zn) and chromium (Cr). Limit values for AgroOrganic and Agro certification category in the European Biochar Certificate (EBC).*[8]

	**Landscaping wood [mg kg^{-1}]**	**Landscaping wood + ash [mg kg^{-1}]**	**EBC AgroOrganic [mg kg^{-1}]**	**EBC Agro [mg kg^{-1}]**
**As**	1.3	1.8	13	13
**Pb**	6.0	8.0	45	120
**Cd**	<0.2	<0.3	0.7	1.5
**Cu**	19.0	71.0	70	100
**Ni**	6.0	10.0	25	50
**Hg**	<0.07	<0.07	0.4	1.0
**Zn**	79.0	90.0	200	400
**Cr**	9.0	26.0	70	90

Table S8: *Contents of the 16 polycyclic aromatic hydrocarbons (PAH) prioritized by the Environmental Protection Agency (EPA) of the United States and the 8 PAH prioritized by the European Food Safety Authority (EFSA). Contents are given excluding the limit of detection (LOD) of the analysis method. Limit values in the European Biochar Certificate (EBC) are 6 mg kg^{-1} for 16 EPA PAH, 1.0 mg kg^{-1} for 8 EFSA PAH, and each 1 mg kg^{-1} for Benzo[e]pyrene and Benzo[j]fluoranthene, the latter two have not been quantified in the present study.*[8]

		mg kg^{-1}	Landscaping wood	Landscaping wood + ash
16 EPA PAH		Naphtalene	3.2 ± 0.2	3.1 ± 0.4
16 EPA PAH		Acenaphthylene	<0.1	<0.1
16 EPA PAH		Acenaphthene	0.1 ± 0.0	0.2 ± 0.0
16 EPA PAH		Fluorene	0.7 ± 0.1	0.7 ± 0.1
16 EPA PAH		Phenanthrene	1.0 ± 0.1	1.0 ± 0.0
16 EPA PAH		Anthracene	0.3 ± 0.0	0.4 ± 0.0
16 EPA PAH		Fluoranthene	0.3 ± 0.1	0.3 ± 0.0
16 EPA PAH		Pyrene	0.4 ± 0.1	0.5 ± 0.0
16 EPA PAH	8 EFSA PAH	Benzo[*a*]anthracene	0.1 ± 0.0	0.2 ± 0.0
16 EPA PAH	8 EFSA PAH	Chrysene	0.1 ± 0.0	0.1 ± 0.0
16 EPA PAH	8 EFSA PAH	Benzo[*b*]fluoranthene	<0.1	<0.1
16 EPA PAH	8 EFSA PAH	Benzo[*k*]fluoranthene	<0.1	<0.1
16 EPA PAH	8 EFSA PAH	Benzo[*a*]pyrene	<0.1	0.1 ± 0.0
16 EPA PAH	8 EFSA PAH	Indeno[*1,2,3-cd*]pyrene	<0.1	<0.1
16 EPA PAH	8 EFSA PAH	Dibenzo[*ah*]anthracene	<0.1	<0.1
16 EPA PAH	8 EFSA PAH	Benzo[*ghi*]perylene	<0.1	<0.1
		16 EPA PAH excl. LOD	6.2 ± 0.3	6.8 ± 0.7
		8 EFSA PAH excl. LOD	0.2 ± 0.1	0.4 ± 0.1

References

(1) DIN EN ISO 10390:2022-08, Boden, Behandelter Bioabfall Und Schlamm_- Bestimmung Des pH-Werts (ISO_10390:2021); Deutsche Fassung EN_ISO_10390:2022. https://doi.org/10.31030/3226922.

(2) DIN EN 15169:2007-05, Charakterisierung von Abfall_- Bestimmung Des Glühverlustes in Abfall, Schlamm Und Sedimenten; Deutsche Fassung EN_15169:2007. https://doi.org/10.31030/9771848.

(3) DIN EN ISO 17294-2:2024-03, Wasserbeschaffenheit - Anwendung Der Induktiv Gekoppelten Plasma-Massenspektrometrie (ICP-MS) - Teil 2: Bestimmung von Ausgewählten Elementen Einschließlich Uran-Isotope (ISO 17294-2:2023); Deutsche Fassung EN ISO 17294-2:2023, 2024. https://doi.org/10.31030/3503779.

(4) DIN EN ISO 12846:2012-08, Wasserbeschaffenheit_- Bestimmung von Quecksilber_- Verfahren Mittels Atomabsorptionsspektrometrie (AAS) Mit Und Ohne Anreicherung (ISO_12846:2012); Deutsche Fassung EN_ISO_12846:2012. https://doi.org/10.31030/1869854.

(5) DIN EN ISO 16948:2015-09, Biogene Festbrennstoffe - Bestimmung Des Gesamtgehaltes an Kohlenstoff, Wasserstoff Und Stickstoff (ISO 16948:2015); Deutsche Fassung EN ISO 16948:2015, 2015. https://doi.org/10.31030/2244183.

(6) DIN e.V. DIN EN ISO 18122:2023-02, Biogene Festbrennstoffe_- Bestimmung Des Aschegehaltes (ISO_18122:2022); Deutsche Fassung EN_ISO_18122:2022, 2023. https://doi.org/10.31030/3379889.

(7) Verband Deutscher Landwirtschaftlicher Untersuchungs- und Forschungs- anstalten. *VDLUFA Method 6.5.3*; VDLUFA-Verlag: Speyer, 2012; Vol. Volume III.

(8) EBC 2012-2024. "European Biochar Certificate - Guidelines for a Sustainable Production of Biochar." Carbon Standards International (CSI), Frick, Switzerland. (Http://European-Biochar.Org). Version 10.4 from 20th Dec 2024, 2024.

(9) DIN EN 16318:2016-07, Düngemittel Und Kalkdünger_- Bestimmung von Chrom(VI) Mit Photometrie (Verfahren_A) Und Mit Ionenchromatographie Mit Spektrometrischer Detektion (Verfahren_B); Deutsche Fassung EN_16318:2013+A1:2016. https://doi.org/10.31030/2415710.

(10) Bachmaier, H.; Kuptz, D.; Hartmann, H. Wood Ashes from Grate-Fired Heat and Power Plants: Evaluation of Nutrient and Heavy Metal Contents. *Sustainability* **2021**, *13* (10), 5482. https://doi.org/10.3390/su13105482.

(11) Mayer, E.; Eichermüller, J.; Endriss, F.; Baumgarten, B.; Kirchhof, R.; Tejada, J.; Kappler, A.; Thorwarth, H. Utilization and Recycling of Wood Ashes from Industrial Heat and Power Plants Regarding Fertilizer Use. *Waste Management* **2022**, *141*, 92–103. https://doi.org/10.1016/j.wasman.2022.01.027.

(12) Olanders, B.; Steenari, B.-M. Characterization of Ashes from Wood and Straw. *Biomass and Bioenergy* **1995**, *8* (2), 105–115. https://doi.org/10.1016/0961-9534(95)00004-Q.

(13) European Union. *REGULATION (EU) 2019/1009 OF THE EUROPEAN PARLIAMENT AND OF THE COUNCIL of 5 June 2019*; 2019. https://eur-lex.europa.eu/legal-content/EN/TXT/?uri=CELEX%3A02019R1009-20241120 (accessed 2025-03-06).

(14) BGH. *Bundesgütegemeinschaft Holzasche: Verwertung von Holzaschen*. URL Accessed on 18.07.2021. https://www.kompost.de/fileadmin/docs/Archiv/Thema_Position/5.3.2_Thema_Verwertung_von_Holzaschen_2013-final.pdf (accessed 2021-02-08).

(15) Bundesministerium für Land- und Forstwirtschaft, Umwelt und Wasserwirtschaft; Fachbeirat für Bodenfruchtbarkeit und Bodenschutz. *Richtlinie Für Den Sachgerechten Einsatz von Pflanzenaschen Zur Verwertung Auf Land- Und Fortstwirtschaftlich Genutzten*

Flächen.; 2011. https://www.ages.at/download/sdl-
eyJ0eXAiOiJKV1QiLCJhbGciOiJIUzI1NiJ9.eyJpYXQiOjE2MDk0NTkyMDAsImV4cCI6N
DA3MDkwODgwMCwidXNlciI6MCwiZ3JvdXBzIjpbMCwtMV0sImZpbGUiOiJmaWxlYW
RtaW4vQUdFU18yMDIyLzVfVU1XRUxUL1JhZGlvYWt0aXZpdFx1MDBlNHQvUmFka
W9ha3Rpdml0XHUwMGU0dF9pbl9kZXJfVW13ZWx0L1JpY2h0bGluaWVfZlx1MDBmY3
Jfc2FjaGdlcmVjaHRlbl9FaW5zYXR6X3Zvbl9QZmxhbnplbmFzY2hlbi5wZGYiLCJwYWdl
IjoxMjg0fQ.NWYqX15H8CdyRI0VdqxtlYP4-5YWKqSVHtI-
RAxn9PE/Richtlinie_für_sachgerechten_Einsatz_von_Pflanzenaschen.pdf (accessed 2024-
09-13).
